# Magnetic Hysteresis at 10 K in Single Molecule Magnet Self‐Assembled on Gold

**DOI:** 10.1002/advs.202000777

**Published:** 2021-01-21

**Authors:** Chia‐Hsiang Chen, Lukas Spree, Emmanouil Koutsouflakis, Denis S. Krylov, Fupin Liu, Ariane Brandenburg, Georgios Velkos, Sebastian Schimmel, Stanislav M. Avdoshenko, Alexander Fedorov, Eugen Weschke, Fadi Choueikani, Philippe Ohresser, Jan Dreiser, Bernd Büchner, Alexey A. Popov

**Affiliations:** ^1^ Leibniz Institute for Solid State and Materials Research Helmholtzstraße 20 Dresden 01069 Germany; ^2^ Department of Medicinal and Applied Chemistry Kaohsiung Medical University Kaohsiung 807 Taiwan; ^3^ Center for Quantum Nanoscience Institute for Basic Science (IBS) Seoul 03760 Republic of Korea; ^4^ Helmholtz‐Zentrum Berlin für Materialien und Energie Wilhelm‐Conrad‐Röntgen‐Campus BESSY II Albert‐Einstein‐Strasse 15 Berlin D‐12489 Germany; ^5^ Synchrotron SOLEIL L'Orme des Merisiers Saint‐Aubin, BP 48 Gif‐sur‐Yvette 91192 France; ^6^ Swiss Light Source Paul Scherrer Institute Villigen PSI CH‐5232 Switzerland

**Keywords:** fullerenes, magnetic hysteresis, self‐assembled monolayers, single‐molecule magnets, XMCD

## Abstract

Tremendous progress in the development of single molecule magnets (SMMs) raises the question of their device integration. On this route, understanding the properties of low‐dimensional assemblies of SMMs, in particular in contact with electrodes, is a necessary but difficult step. Here, it is shown that fullerene SMM self‐assembled on metal substrate from solution retains magnetic hysteresis up to 10 K. Fullerene‐SMM DySc_2_N@C_80_ and Dy_2_ScN@C_80_ are derivatized to introduce a thioacetate group, which is used to graft SMMs on gold. Magnetic properties of grafted SMMs are studied by X‐ray magnetic circular dichroism and compared to the films of nonderivatized fullerenes prepared by sublimation. In self‐assembled films, the magnetic moments of the Dy ions are preferentially aligned parallel to the surface, which is different from the disordered orientation of endohedral clusters in nonfunctionalized fullerenes. Whereas chemical derivatization reduces the blocking temperature of magnetization and narrows the hysteresis of Dy_2_ScN@C_80_, for DySc_2_N@C_80_ equally broad hysteresis is observed as in the fullerene multilayer. Magnetic bistability in the DySc_2_N@C_80_ grafted on gold is sustained up to 10 K. This study demonstrates that self‐assembly of fullerene‐SMM derivatives offers a facile solution‐based procedure for the preparation of functional magnetic sub‐monolayers with excellent SMM performance.

## Graphical abstract

Fullerene single molecule magnets DySc_2_N@C_80_ and Dy_2_ScN@C_80_ are derivatized with thioacetate linker groups and assembled as monolayers on the Au(111) substrate. Magnetic properties of the self‐assembled monolayers are studied by X‐ray magnetic circular dichroism. The molecules retain their magnetic hysteresis on gold, which for DySc_2_N@C_80_ is observed up to 10 K.

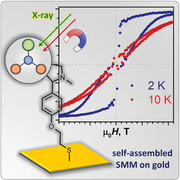

Since their first discovery in 1993,^[^ ^1^ ^]^ single molecule magnets (SMMs), i.e., molecular materials with bistable magnetic ground state and slow relaxation of magnetization, have been seen as building elements for nanoscale spintronic devices.^[^ ^2^ ^]^ Realization of spin valves, giant magnetoresistance, and coherent spin manipulation in single TbPc_2_ molecules attached to carbon nanotubes at sub‐Kelvin temperatures demonstrate the great potential of molecular spintronics with SMMs.^[^ ^3^ ^]^ A prerequisite for potential nanodevice implementation of a given SMM is that its magnetic bistability demonstrated in bulk powder or crystal sample should be preserved on a substrate, which will serve as an electrode of a device.^[^ ^4^ ^]^ Thus, surface deposition is a necessary and hardly avoidable step if the truly single‐molecule nature of SMMs is to be explored and utilized.^[^ ^2^, ^5^ ^]^

Whereas development of better and stronger SMMs over the last decade was tremendous,^[^ ^6^ ^]^ the magnetic bistability of SMMs on surfaces is still rather modest in comparison to bulk SMMs and surface atomic systems. The benchmarks for the sub‐monolayers of discrete magnetic units on surfaces are set so far by single atomic magnets such as Ho atoms on MgO|Ag(100) with magnetic bistability up to 30 K.^[^ ^7^ ^]^ However, processability of single atomic systems outside of an ultrahigh‐vacuum chamber is a rather complicated issue. Molecular materials can be processed more easily, and offer chemical routes to surface grafting via solution‐based self‐assembly. Yet, the studies of the low‐dimensional assemblies of SMMs are lagging behind, which is in part caused by the limited stability of many SMM preventing their sublimation and complicating further chemical modification for surface grafting. The best on‐surface SMM properties to date have been demonstrated for sublimation‐deposited sub‐monolayers of TbPc_2_ with magnetic bistability up to 8–9 K on MgO|Ag(100)^[^ ^8^ ^]^ and graphene/SiC(0001)^[^ ^9^ ^]^ substrates.

Fullerenes as carbon cages with empty voids capable of storing magnetic clusters offer important advantages on this route.^[^ ^10^ ^]^ On the one hand, the magnetism of endohedral species can be tuned in a broad range by judicious choice of encapsulated metals and their combinations.^[^ ^11^ ^]^ On the other hand, the carbon cage is very stable thermally and chemically, which enables sublimation^[^ ^12^ ^]^ or further chemical functionalization^[^ ^13^ ^]^ of endohedral metallofullerenes (EMFs). In this work we use these advantages to prepare self‐assembled sub‐monolayer films (SAMs) of fullerenes DySc_2_N@C_80_ and Dy_2_ScN@C_80_ on Au(111) and achieve the highest temperature of magnetic hysteresis for SMMs on a metallic substrate.

Due to the close distance to the central nitride ion, Dy ions in both EMFs have strong magnetic anisotropy. The two molecules are different only in the number of Dy ions in the endohedral trimetal cluster, which however has a strong influence on their low‐temperature SMM behavior. DySc_2_N@C_80_ shows quantum tunneling of magnetization (QTM) near zero field and has a blocking temperature of magnetization (*T*_B_) near 7 K,^[^ ^14^ ^]^ whereas intramolecular dipolar and exchange interactions between Dy ions in Dy_2_ScN@C_80_ quench the QTM and shift the *T*_B_ to 8 K.^[^ ^15^ ^]^ To graft these EMFs to gold substrates, they were first functionalized with a thioacetate (SAc) linker via 1,3‐dipolar cycloaddition (**Figure** **1**) using a modification of the procedure described earlier for C_60_.^[^ ^16^ ^]^ In the original procedure in ref. ^[^ ^16^ ^]^, C_60_ was functionalized with a thiol linker. However, EMFs require higher reaction temperature, and direct thiol attachment to EMFs was found unstable, requiring the use of a more stable thioacetate linker instead. Cycloaddition reactions were performed for three M_3_N@C_80_ molecules (M_3_N = Sc_3_N, DySc_2_N, and Dy_2_ScN) giving pure derivatives (denoted as **M_3_N‐RSAc** hereafter) after chromatographic separation. Further details and characterization of the products are given in ESI (Figures S1–S7, Supporting Information).

**Figure 1:**
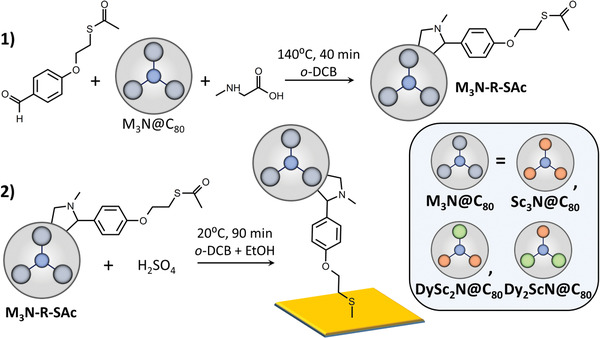
Derivatization of fullerene M_3_N@C_80_ with a thioacetate group (M_3_N = Sc_3_N, DySc_2_N, and Dy_2_ScN) via 1,3‐dipolar cycloaddition (1) and deprotection of **M_3_N‐R‐SAc** by acid treatment in *o*‐DCB/ethanol leading to the formation of **M_3_N‐SAM** with partially chemisorbed and partially physisorbed molecules on gold (2).

SQUID (superconducting quantum interference device) magnetometry proved that the fullerenes retained their SMM behavior after derivatization. **DySc_2_N‐RSAc** has similarly broad hysteresis as the pristine DySc_2_N@C_80_, whereas its *T*_B_ is increased to 8 K (Figure S7, Supporting Information). For Dy_2_ScN@C_80_ the cycloaddition resulted in a narrower hysteresis and a reduced *T*_B_ of 4 K in **Dy_2_ScN‐RSAc** (Figure S8, Supporting Information). These findings agree well with the earlier results on fullerene cycloadducts.^[^ ^17^ ^]^ The different influence of cycloaddition on the hysteretic behavior of DySc_2_N@C_80_ and Dy_2_ScN@C_80_ is not very clear. Presumably, the main reason is the different mechanism of the magnetization relaxation in two SMMs at low temperature. Whereas in‐field relaxation of magnetization in single‐on magnet DySc_2_N@C_80_ is governed by the Raman mechanism,^[^ ^14a^ ^]^ in Dy_2_ScN@C_80_ the relaxation follows the Orbach mechanism via the state with antiferromagnetic coupling of Dy moments.^[^ ^15b^ ^]^ The latter process may be more susceptible to the changes of vibrational density of states in the molecules when the internal motion of the cluster is hindered by the cycloadduct.

The previous attempt of obtaining EMF‐SMM monolayers with thioether linker –S–CH_3_ in a similar molecular arrangement resulted in a low coverage of physisorbed molecules.^[^ ^17a^ ^]^ In this work, chemisorption (Figure 1) was attempted following ref. ^[^ ^18^ ^]^ by dissolving **M_3_N‐RSAc** derivatives in *o*‐dichlorobenzene (DCB)/ethanol (10:1) and adding H_2_SO_4_ in the presence of Au(111)/mica substrates (PHASIS, Switzerland). After incubation in solution for 90 min, the substrates were washed with an excess of *o*‐DCB followed by ethanol and dried in a flow of nitrogen. Formation of self‐assembled films with sub‐monolayer coverage (**M_3_N‐SAMs** hereafter) was verified by XPS (X‐ray photoelectron spectroscopy; Figure S9, Supporting Information) and X‐ray absorption spectroscopy (XAS, see below). XPS spectra in the S 2p range revealed the presence of Au‐bonded sulfur at 161.1 eV, nonbonded sulfur at 162–163 eV and a broad signal of oxidized sulfur at 169 eV (Figure S9, Supporting Information). The oxidized sulfur likely originates form the traces of the sulfuric acid used in the deposition procedure, which is in line with the higher sulfur content than expected for the chemical composition of the functionalized fullerenes. Considering that Au‐bonded and nonbonded sulfur originates from chemi‐ and physisorbed molecules, the former constitute ≈40% in **DySc_2_N‐SAM** and 30% in **Dy_2_ScN‐SAM**. The SAMs prepared from C_60_ functionalized with thioacetate groups by Tour et al.^[18a]^ exhibited similar XPS spectra with bonded and nonbonded sulfur. Since ellipsometry analysis proved formation of monolayers in their samples, the authors concluded that functionalized C_60_ molecules may pack in a head‐to‐tail manner on the surface. This contamination precluded complete monolayer coverage of the metal surface with fullerenes, thus giving sub‐monolayer films as a result of solution‐based self‐assembly. XAS analysis of our samples (see below) also excludes formation of multilayers, and we thus conclude that our sub‐monolayer **M_3_N‐SAMs** consist of a mixture of chemi‐ and physisorbed molecules, for which head‐to‐tail arrangement may be a reasonable possibility.

Structural and magnetic properties of the SAMs were then studied by X‐ray natural linear dichroism (XNLD) and X‐ray magnetic circular dichroism (XMCD) at the Dy‐*M*_4,5_ absorption edge. For each sample, we first discuss the XNLD and XMCD spectra and their angular dependence to establish possible ordering of fullerene molecules on the surface, and then proceed to the description of the hysteretic behavior. As a reference, XAS measurements were also performed for the films of nonfunctionalized fullerenes on Au(111) prepared by vacuum evaporation.

XAS studies of **Dy_2_ScN‐SAM** were performed at the UE46_PGM‐1 beamline^[^ ^19^ ^]^ at BESSY II (Helmholtz‐Zentrum Berlin) and the X‐Treme beamline at the Swiss Light Source (Paul Scherrer Institute).^[^ ^20^ ^]^ XNLD and XMCD experiments at two beamlines with fresh **Dy_2_ScN‐SAM** samples prepared immediately before the measurements demonstrated good reproducibility of the procedure (Figure S10, Supporting Information). Total XAS intensity of **Dy_2_ScN‐SAM** is only slightly higher than that of the evaporated sub‐monolayer with ca 0.5 monolayer coverage (**Figure** **2**a,b; Figures S11 and S12, Supporting Information), proving that the deposition procedure did not form multilayers and is limited to a sub‐monolayer coverage. At room temperature, a Dy‐*M*_5_ XNLD signal of 13% of the XAS maximum was observed indicating partial ordering of the Dy_2_ScN clusters in the SAM (Figure S10, Supporting Information; see also Figure S13 (Supporting Information) for simulations of XAS and XNLD spectra with the code MULTIX^[^ ^21^ ^]^). Low temperature XMCD measurements at 2–5 K also confirmed this observation. Figure 2a shows XAS and XMCD spectra at the Dy‐*M*_5_ edge in two orientations of the beam and magnetic field versus the surface. The XMCD signal at grazing incidence (30°) is noticeably stronger than for the normal incidence (90°) indicating that the magnetic moments of Dy^3+^ ions tend to be parallel to the surface. Importantly, the ordering effect in the **Dy_2_ScN‐SAM** is more pronounced that in the sub‐monolayer of nonfunctionalized Dy_2_ScN@C_80_ (Figure 2b, see also Figures S14–S16 (Supporting Information) and ref. ^[^ ^22^ ^]^ for more details). Note also that earlier we could not observe ordering for thioether‐functionalized Dy_2_ScN@C_80_ physisorbed on Au(111).^[^ ^17^, ^23^ ^]^ Thus, cycloaddition to the fullerene cage freezes rotation of the endohedral cluster inside the fullerene, whereas the self‐assembly hinders rotation of the molecules on the surface. Angular dependence of magnetization is also evident from magnetic hysteresis measurements (Figure 2c). At 2 K, **Dy_2_ScN‐SAM** demonstrates magnetic hysteresis at both incidence angles of 30° and 90°. In line with the SQUID measurements of powder samples (Figure S8, Supporting Information), the opening of the hysteresis for **Dy_2_ScN‐SAM** is narrower than for the sub‐monolayer of Dy_2_ScN@C_80_ (Figure 2d).

**Figure 2:**
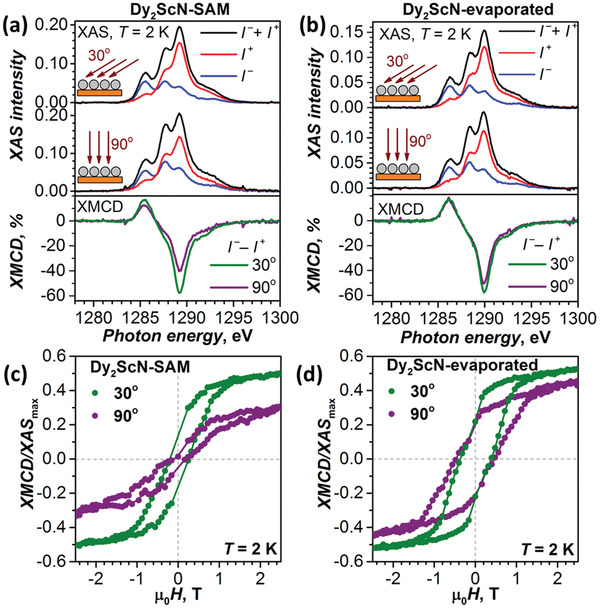
XAS and XMCD spectra of Dy_2_ScN‐SAM a) and evaporated Dy_2_ScN@C_80_ sub‐monolayer b) on Au(111) measured at 30° and 90° orientation of the X‐ray and magnetic field versus the surface; *T* ≈ 2 K, *H* = 6.5 T, only the Dy‐*M*_5_ edge is shown (see the Supporting Information for the whole Dy‐*M*_4,5_ range). X‐ray polarizations are denoted at *I* ^+^ and *I* ^−^, nonpolarized XAS is a sum of *I* ^+^ and *I* ^−^, and XMCD is their difference normalized to the maximum of XAS. Magnetic hysteresis of Dy_2_ScN‐SAM c) and evaporated Dy_2_ScN@C_80_ sub‐monolayer d) on Au(111) measured by XMCD technique for two orientations of the sample; *T* ≈ 2 K, sweep rate 2 T min^−1^; dots are experimental values, lines are added to guide the eye. The measurements of the evaporated sub‐monolayer are from ref. ^[^ ^22^ ^]^.

Because of the considerable remanence and coercivity found in its powder samples,^[^ ^15^ ^]^ Dy_2_ScN@C_80_ was in focus of the earlier surface studies of EMF‐SMMs, and magnetic properties of its monolayers were studied by XMCD on Rh(111), *h*‐BN|Rh(111), Au(111), Ag(100), and MgO(10 ML)|Ag(100).^[^ ^12^ ^]^ All of them exhibited magnetic hysteresis near 2 K, but none retained magnetic bistability at 6 K. Meanwhile, it appears that the magnetism of the dinuclear Dy_2_ScN cluster is more susceptible to the external effects than that of the mononuclear DySc_2_N. As described above, exohedral functionalization of the fullerene decreases the temperature range of magnetic bistability of Dy_2_ScN@C_80_, but improves the SMM behavior of DySc_2_N@C_80_.^[^ ^17a^ ^]^ Likewise, DySc_2_N@C_80_ retains magnetic hysteresis inside carbon nanotubes, whereas the Dy_2_ScN@C_80_‐based peapod does not show magnetic hysteresis.^[^ ^24^ ^]^ Below we demonstrate that SMM behavior of a DySc_2_N@C_80_ monolayer on a metallic substrate is also more robust than that of Dy_2_ScN@C_80_.

XAS and XMCD studies of **DySc_2_N‐SAM** were performed at the DEIMOS beamline at synchrotron SOLEIL.^[^ ^25^ ^]^ In agreement with the molecular composition, XAS intensity of **DySc_2_N‐SAM** is twice smaller than that of **Dy_2_ScN‐SAM** (**Figure** **3**), which also proves similar sub‐monolayer coverage of the gold surface by fullerene molecules in both SAMs. At room temperature **DySc_2_N‐SAM** showed XNLD signal with the relative intensity of 7%, which increased to 8% upon lowering temperature to 2 K (Figure S17, Supporting Information). A noticeable angular dependence can be also seen in the XMCD spectra measured between 30° and 90° (Figure 3; Figure S18, Supporting Information). The XMCD signal increases gradually with the decrease of the incidence angle from 90° to 30° (Figure 3c), showing that the orientation of the DySc_2_N cluster parallel to the surface is more preferable.

**Figure 3:**
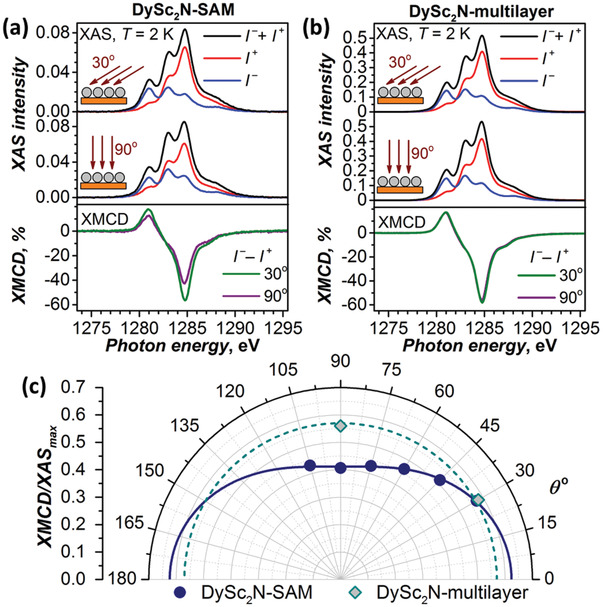
XAS and XMCD spectra of DySc_2_N‐SAM a) and evaporated DySc_2_N@C_80_ multilayer b) on Au(111) measured at 30° and 90° orientations of the X‐ray and magnetic field versus the surface; *T* ≈ 2 K, *H* = 6 T, only the Dy‐*M*_5_ edge is shown (see the Supporting Information for the whole Dy‐*M*_4,5_ range). c) Angular dependence of XMCD asymmetry for DySc_2_N‐SAM on Au(111). *θ* is defined as the angle between X‐ray beam/magnetic field and the surface, dots are experimental values, solid line is a fit with the function XMCD/XAS  =  *C*_1_cos ^2^(*θ*) + *C*_2_. Dashed line shows the isotopic distribution for a completely disordered cluster ( *C*_1_ =  0) such as found for the DySc_2_N@C_80_ multilayer.

As a reference, a multilayer film (5–6 monolayers) of nonfunctionalized DySc_2_N@C_80_ was prepared by evaporation onto an Au(111) single crystal. For such a film, XMCD does not show any angular dependence (Figure 3; Figure S19, Supporting Information), proving that the endohedral cluster is completely disordered, although carbon cages form ordered closed‐packed hexagonal layers as can be seen in the STM topography (Figures S20 and S21, Supporting Information).

**Figure** **4**a shows that at 2 K **DySc_2_N‐SAM** has an open magnetic hysteresis between −2 T and 2 T. Near zero field the hysteresis tends to close due to the fast relaxation of magnetization via QTM as also observed in the bulk powder sample (Figure S7, Supporting Information). Hysteresis of a DySc_2_N@C_80_ multilayer at 2 K is similarly broad, but demonstrates noticeable deviations from **DySc_2_N‐SAM** in the field less than 1 T (Figure 4b). Note that due to the peculiarities of the total electron yield detection mode, the XMCD intensity during the field sweep shows erratic oscillations at small magnetic fields, which prevents an accurate measurement of the hysteresis shape close to 0 T. Very recent sub‐Kelvin study of {Fe_4_} molecular magnet deposited on superconducting Pb(111) surface showed that this problem can be mitigated by very low sweep rate and continuous switching of the X‐ray beam polarity,^[^ ^26^ ^]^ and the use of this approach seems promising in future studies of QTM in EMF monolayers.

**Figure 4:**
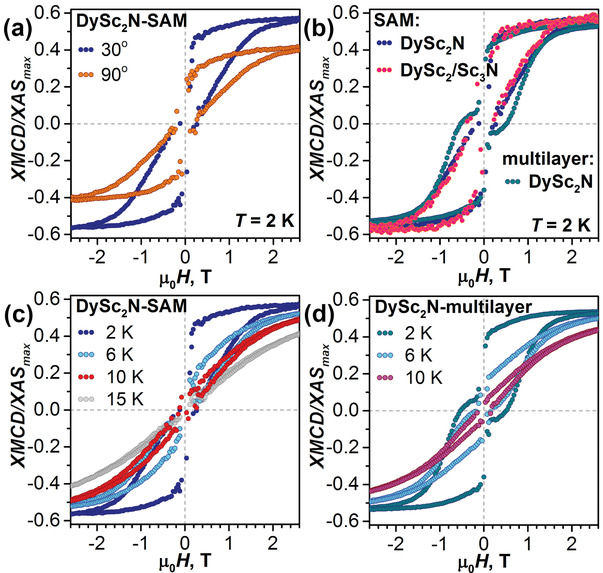
Magnetic hysteresis curves measured by XMCD technique, sweep rate 2 T min^−1^. a) DySc_2_N‐SAM measured at 30° and 90°; *T* ≈ 2 K; b) DySc_2_N‐SAM, diluted DySc_2_/Sc_3_N‐SAM, and DySc_2_N@C_80_ multilayer measured at 30°, *T* ≈ 2 K. c) DySc_2_N‐SAM measured at 30° at different temperatures; d) DySc_2_N@C_80_ multilayer on Au(111) measured at 30° at different temperatures.

The QTM of DySc_2_N@C_80_ strongly depends on the molecular environment and can be reduced significantly by magnetic dilution.^[^ ^14^, ^17^ ^]^ To find if moderate dilution can have a similar effect on surface, a SAM was prepared from a 1:4 mixture of **DySc_2_N‐RSAc** and **Sc_3_N‐RSAc**. Dy‐*M*_4,5_ XAS intensity of **DySc_2_/Sc_3_N‐SAM** is decreased according to the four‐fold dilution (Figures S22 and S23, Supporting Information), but the shape of the hysteresis remains virtually identical to that of **DySc_2_N‐SAM** (Figure 4b). Unfortunately, studies of a stronger on‐surface dilution are limited by a decreasing signal‐to‐noise ratio.

Magnetization curves of **DySc_2_N‐SAM** and DySc_2_N@C_80_ multilayer were then measured at different temperatures. Figure 4c demonstrates that the hysteresis in **DySc_2_N‐SAM** is getting narrower with the temperature increase, but still remains open at 10 K (see also Figure S24 in the Supporting Information for more temperatures). Thus, the surface grafting is not deteriorating SMM properties of the DySc_2_N@C_80_ derivative. However, a direct comparison of the monolayer XMCD studies to the results of SQUID magnetometry for powder samples may appear not fully representative because of the X‐ray demagnetization effect^[^ ^27^ ^]^ and other differences in the measurement conditions, such as the magnetic field sweep rate. A bulletproof evidence is provided by the comparison between **DySc_2_N‐SAM** and DySc_2_N@C_80_ multilayer, representing the properties of the bulk fullerene material. Similar hysteretic behavior found by XMCD for the two samples (compare Figure 4c,d) proves that the direct contact with the metallic substrate does not affect the magnetic bistability of DySc_2_N@C_80_ in the SAM. This is different from TbPc_2_ on Au(111), which exhibits open hysteresis up to 15 K in a multilayer, but shows no hysteresis in the monolayer at 8 K.^[^ ^28^ ^]^

To conclude, we have functionalized metallofullerene‐SMMs DySc_2_N@C_80_ and Dy_2_ScN@C_80_ with surface‐anchoring groups and prepared self‐assembled sub‐monolayer films thereof on Au(111) from solution. Chemical functionalization is found to influence the SMM properties of both fullerenes, and appears to be beneficial for DySc_2_N@C_80_, which increases the blocking temperature of magnetization in the cycloadduct. Most importantly, XMCD studies of SAMs revealed that the metallic substrate does not impose a considerable influence on the SMM behavior of surface‐grafted fullerenes, which is attributed to the shielding of magnetic units by the carbon cage. This allows the self‐assembled sub‐monolayer of the DySc_2_N@C_80_ derivative to exhibit the highest temperature of hysteresis yet observed in SMM monolayers on surfaces proving its high potential for the exploration of SMM‐based spintronic devices. In the future, further optimization of the deposition procedure may be required to maximize the ratio of chemisorbed molecules.

## Conflict of Interest

The authors declare no conflict of interest.

## Supporting information

Supporting Information

## References

[1] R. Sessoli , D. Gatteschi , A. Caneschi , M. A. Novak , Nature 1993, 365, 141.

[2] a) C. Cervetti , E. Heintze , L. Bogani , Dalton Trans. 2014, 43, 4220; 24514949 10.1039/c3dt52650j

[3] a) E. Moreno‐Pineda , C. Godfrin , F. Balestro , W. Wernsdorfer , M. Ruben , Chem. Soc. Rev. 2018, 47, 501; 29147698 10.1039/c5cs00933b

[4] a) M. Mannini , F. Pineider , C. Danieli , F. Totti , L. Sorace , P. Sainctavit , M. A. Arrio , E. Otero , L. Joly , J. C. Cezar , A. Cornia , R. Sessoli , Nature 2010, 468, 417; 20981008 10.1038/nature09478

[5] a) A. Cornia , M. Mannini , P. Sainctavit , R. Sessoli , Chem. Soc. Rev. 2011, 40, 3076; 21403949 10.1039/c0cs00187b

[6] a) J.‐L. Liu , Y.‐C. Chen , M.‐L. Tong , Chem. Soc. Rev. 2018, 47, 2431; 29492482 10.1039/c7cs00266a

[7] F. Donati , S. Rusponi , S. Stepanow , C. Wäckerlin , A. Singha , L. Persichetti , R. Baltic , K. Diller , F. Patthey , E. Fernandes , J. Dreiser , Ž. Šljivančanin , K. Kummer , C. Nistor , P. Gambardella , H. Brune , Science 2016, 352, 318. 27081065 10.1126/science.aad9898

[8] C. Wäckerlin , F. Donati , A. Singha , R. Baltic , S. Rusponi , K. Diller , F. Patthey , M. Pivetta , Y. Lan , S. Klyatskaya , M. Ruben , H. Brune , J. Dreiser , Adv. Mater. 2016, 28, 5195. 27159732 10.1002/adma.201506305

[9] G. Serrano , E. Velez‐Fort , I. Cimatti , B. Cortigiani , L. Malavolti , D. Betto , A. Ouerghi , N. B. Brookes , M. Mannini , R. Sessoli , Nanoscale 2018, 10, 2715. 29372744 10.1039/c7nr08372f

[10] a) A. A. Popov , Endohedral Fullerenes: Electron Transfer and Spin, Springer International Publishing, Cham, Switzerland 2017;

[11] a) L. Spree , A. A. Popov , Dalton Trans. 2019, 48, 2861; 30756104 10.1039/c8dt05153d PMC6394203

[12] a) T. Greber , A. P. Seitsonen , A. Hemmi , J. Dreiser , R. Stania , F. Matsui , M. Muntwiler , A. A. Popov , R. Westerström , Phys. Rev. Mater. 2019, 3, 014409;

[13] a) P. Jin , Y. Li , S. Magagula , Z. Chen , Coord. Chem. Rev. 2019, 388, 406;

[14] a) D. Krylov , F. Liu , A. Brandenburg , L. Spree , V. Bon , S. Kaskel , A. Wolter , B. Buchner , S. Avdoshenko , A. A. Popov , Phys. Chem. Chem. Phys. 2018, 20, 11656; 29671443 10.1039/c8cp01608a PMC5933001

[15] a) D. S. Krylov , F. Liu , S. M. Avdoshenko , L. Spree , B. Weise , A. Waske , A. U. B. Wolter , B. Büchner , A. A. Popov , Chem. Commun. 2017, 53, 7901; 10.1039/c7cc03580b PMC5730045 28656179

[16] M. del Carmen Gimenez‐Lopez , M. T. Räisänen , T. W. Chamberlain , U. Weber , M. Lebedeva , G. A. Rance , G. A. D. Briggs , D. Pettifor , V. Burlakov , M. Buck , A. N. Khlobystov , Langmuir 2011, 27, 10977. 21744819 10.1021/la200654n

[17] a) C. H. Chen , D. S. Krylov , S. M. Avdoshenko , F. Liu , L. Spree , R. Westerström , C. Bulbucan , M. Studniarek , J. Dreiser , A. U. B. Wolter , B. Büchner , A. A. Popov , Nanoscale 2018, 10, 11287; 29882575 10.1039/c8nr00511g PMC6018719

[18] a) Y. Shirai , L. Cheng , B. Chen , J. M. Tour , J. Am. Chem. Soc. 2006, 128, 13479; 17031961 10.1021/ja063451d

[19] E. Weschke , E. Schierle , J. Large‐Scale Res. Facil. 2018, 4, A127.

[20] C. Piamonteze , U. Flechsig , S. Rusponi , J. Dreiser , J. Heidler , M. Schmidt , R. Wetter , M. Calvi , T. Schmidt , H. Pruchova , J. Krempasky , C. Quitmann , H. Brune , F. Nolting , J. Synchrotron Radiat. 2012, 19, 661. 22898943 10.1107/S0909049512027847

[21] A. Uldry , F. Vernay , B. Delley , Phys. Rev. B 2012, 85, 125133.

[22] D. S. Krylov , S. Schimmel , V. Dubrovin , F. Liu , T. T. N. Nguyen , L. Spree , C.‐H. Chen , G. Velkos , C. Bulbucan , R. Westerström , M. Studniarek , J. Dreiser , C. Hess , B. Büchner , S. M. Avdoshenko , A. A. Popov , Angew. Chem., Int. Ed. 2020, 59, 5756. 10.1002/anie.201913955 PMC7155138 31860759

[23] S. M. Avdoshenko , J. Comput. Chem. 2018, 39, 1594. 29681135 10.1002/jcc.25231

[24] a) R. Nakanishi , J. Satoh , K. Katoh , H. Zhang , B. K. Breedlove , M. Nishijima , Y. Nakanishi , H. Omachi , H. Shinohara , M. Yamashita , J. Am. Chem. Soc. 2018, 140, 10955; 30125097 10.1021/jacs.8b06983

[25] P. Ohresser , E. Otero , F. Choueikani , K. Chen , S. Stanescu , F. Deschamps , T. Moreno , F. Polack , B. Lagarde , J.‐P. Daguerre , F. Marteau , F. Scheurer , L. Joly , J.‐P. Kappler , B. Muller , O. Bunau , P. Sainctavit , Rev. Sci. Instrum. 2014, 85, 013106. 24517744 10.1063/1.4861191

[26] G. Serrano , L. Poggini , M. Briganti , A. L. Sorrentino , G. Cucinotta , L. Malavolti , B. Cortigiani , E. Otero , P. Sainctavit , S. Loth , F. Parenti , A.‐L. Barra , A. Vindigni , A. Cornia , F. Totti , M. Mannini , R. Sessoli , Nat. Mater. 2020, 19, 546. 32066930 10.1038/s41563-020-0608-9

[27] J. Dreiser , R. Westerström , C. Piamonteze , F. Nolting , S. Rusponi , H. Brune , S. Yang , A. Popov , L. Dunsch , T. Greber , Appl. Phys. Lett. 2014, 105, 032411.

[28] L. Margheriti , D. Chiappe , M. Mannini , P. E. Car , P. Sainctavit , M.‐A. Arrio , F. B. de Mongeot , J. C. Cezar , F. M. Piras , A. Magnani , E. Otero , A. Caneschi , R. Sessoli , Adv. Mater. 2010, 22, 5488. 20949539 10.1002/adma.201003275

[29] S. I. Fedoseenko , I. E. Iossifov , S. A. Gorovikov , J. S. Schmidt , R. Follath , S. L. Molodtsov , V. K. Adamchuk , G. Kaindl , Nucl. Instrum. Methods Phys. Res., Sect. A 2001, 470, 84.

