# Supplementary material for: Magnetic Hysteresis at 10 K in Single Molecule Magnet Self‐Assembled on Gold
Source: Adv Sci (Weinh). 2021 Jan 21;8(5):2000777. doi: 10.1002/advs.202000777 (PMC7927621; doi:10.1002/advs.202000777)
Supplement: Supplementary file 1 — Supporting Information [file ADVS-8-2000777-s001.pdf]

© 2020 Wiley-VCH GmbH

ADVANCED SCIENCE
Open Access

Supporting Information

for *Adv. Sci.,* DOI: 10.1002/advs.202000777

Magnetic hysteresis at 10 K in single molecule magnet self-assembled on gold

Chia-Hsiang Chen, Lukas Spree, Emmanouil Koutsouflakis, Denis S. Krylov, Fupin Liu, Ariane Brandenburg, Georgios Velkos, Sebastian Schimmel, Alexander Fedorov, Eugen Weschke, Fadi Choueikani, Philippe Ohresser, Jan Dreiser, Bernd Büchner, Alexey A. Popov**

Supporting Information

Magnetic hysteresis at 10 K in single molecule magnet self-assembled on gold

Chia-Hsiang Chen, Lukas Spree, Emmanouil Koutsouflakis, Denis S. Krylov, Fupin Liu, Ariane Brandenburg, Georgios Velkos, Sebastian Schimmel, Alexander Fedorov, Eugen Weschke, Fadi Choueikani, Philippe Ohresser, Jan Dreiser, Bernd Büchner, Alexey A. Popov**

Table of contents

Reagents
Synthesis of fullerene derivatives
SQUID magnetometry
Self-assembly from solution and XPS characterization
Details of XAS measurements
XAS/XMCD studies of **Dy_2ScN-SAM** and comparison to evaporated $Dy_2ScN@C_{80}$
STM topography of $Dy_2ScN@C_{80}$ submonolayer
XAS/XMCD studies of **$DySc_2N$-SAM** and comparison to evaporated $DySc_2N@C_{80}$
STM topography of $DySc_2N@C_{80}$ multilayer

Fullerenes and reagents

$Sc_3N@C_{80}$, $DySc_2N@C_{80}$, $Dy_2ScN@C_{80}$ and *p*-[(2-acetylthio)ethoxy]benzaldehyde,[1] were prepared as described in the literature. ^{1}H spectra were obtained on a Bruker Avance spectrometer at 500 MHz. UV-Vis-NIR spectra were recorded from 200 to 2000 nm in toluene by using a 1.0 cm quartz cell with a Shimadzu 3100 spectrophotometer. Matrix-assisted laser desorption ionization (MALDI) mass spectra were recorded on a Bruker autoflex mass spectrometer.

1. (a) *Langmuir* **2011,** *27*, 10977-10985; (b) *Anal. Bioanal. Chem.* **2002**, *373*, 749-753.

Synthesis of $Sc_3N@C_{80}$-R-SAc

$Sc_3N@C_{80}$ (2.9 mg, 2.6 x 10^{-3} mmol), N-methyl glycine (3.5 mg, 0.039 mmol) and *p*-[(2-acetylthio)ethoxy]benzaldehyde (29.3 mg, 0.13 mmol) were placed in an oven-dried 25 mL three-neck round-bottom flask, equipped with a condenser, under a dinitrogen atmosphere. *o*-dichlorobenzene (8 mL) was introduced into the flask via a syringe, and the solution was heated to 140°C for 1 hr. The solution was then cooled to room temperature, the solvent evaporated, and the residue was purified by semi-preparative HPLC with a Buckyprep column (10 × 250 mm), eluting with toluene (flow rate: 1.5 mL min^{-1}). The brown compound **$Sc_3N@C_{80}$-R-SAc** was collected at the retention time of 26 min (Fig. S1a).

^{1}H NMR {500 MHz, CS_2; internal coaxial tube filled with D_6-aceton as lock, 258 K} H 7.2 (d, 1H, aromatic H), 6.45 (m, 2H, aromatic H), 6.14 (d, 1H, aromatic H), 3.64 (d, 1H, -CH_2 pyrrolidine), 3.43 (tq, 2H, -OCH_2), 2.95 (s, 1H, -CH pyrrolidine), 2.60 (s, 2H,-SCH_2), 2.32 (d, 1H, -CH_2 pyrrolidine), 1.84 (s, 3H, -NCH_3), 1.71 (s, 3H,-SCH_3) ppm. MALDI m/z 1360.823 (Fig. S1b)

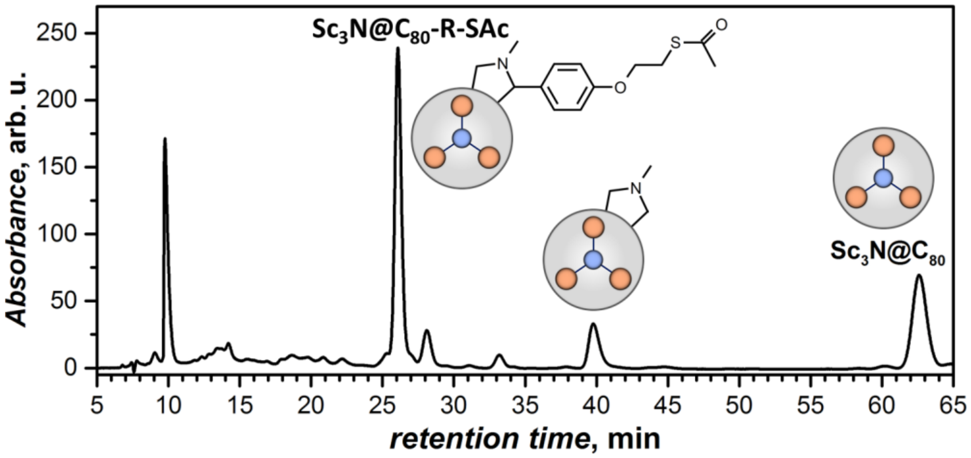

Figure S1a. HPLC trace of the reaction mixture after 1 hour. The peak at 26 min is $Sc_3N@C_{80}$-R-SAc. Pristine (unreacted) $Sc_3N@C_{80}$ elutes at 63 min.

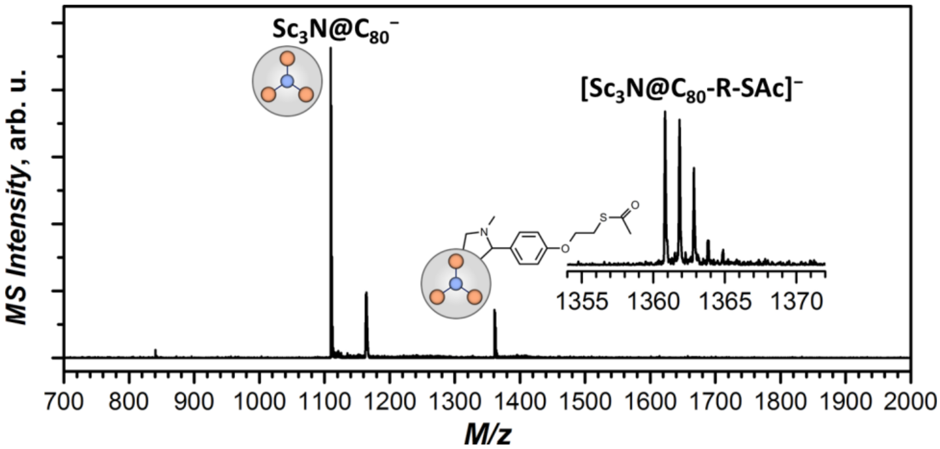

Figure S1b. MALDI-TOF mass-spectrum of the fraction collected at 26 min, proving that it is pure **$Sc_3N@C_{80}$-R-SAc**. The peaks of $Sc_3N@C_{80}^-$ (1108.9 m/z) and $Sc_3N@C_{80}$-C_3H_7N (1165.9 m/z) are due to the fragmentation of **$Sc_3N@C_{80}$-R-SAc** under laser evaporation conditions; since retention times of pristine $Sc_3N@C_{80}$ and $Sc_3N@C_{80}$-C_3H_7N are much longer (Fig. S1a), its presence in the HPLC-separated sample can be excluded.

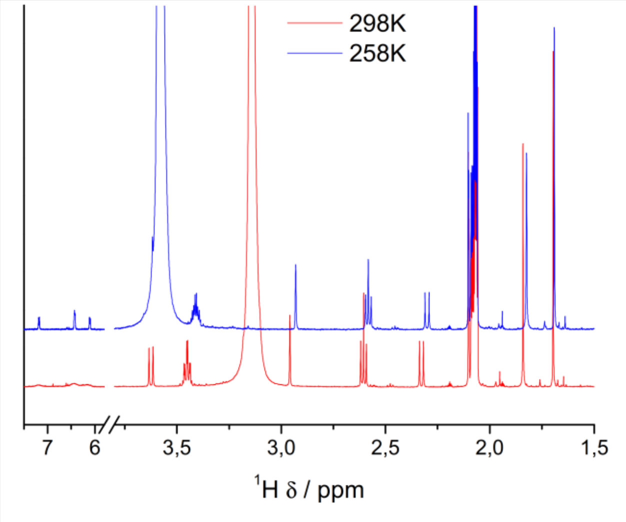

group	δ, ppm @ 258K	intensity
-CO-CH_3	1,71	3
>N-CH_3	1,84	3
-$CH_{2(a)}$-N<	2,32	1
-S-CH_2-	2,60	2
>N-CH<	2,95	1
-O-CH_2-	3,43	2
-$CH_{2(b)}$-N<	3,64	1
aromatic	6,14	1
	6,44	2
	6,45	
	7,2	1

Figure S2. ^{1}H NMR spectra of **$Sc_3N@C_{80}$-R-SAc**. Aromatic protons show noticeable variation with temperature, presumably pointing to the slow rotation on the NMR time scale.

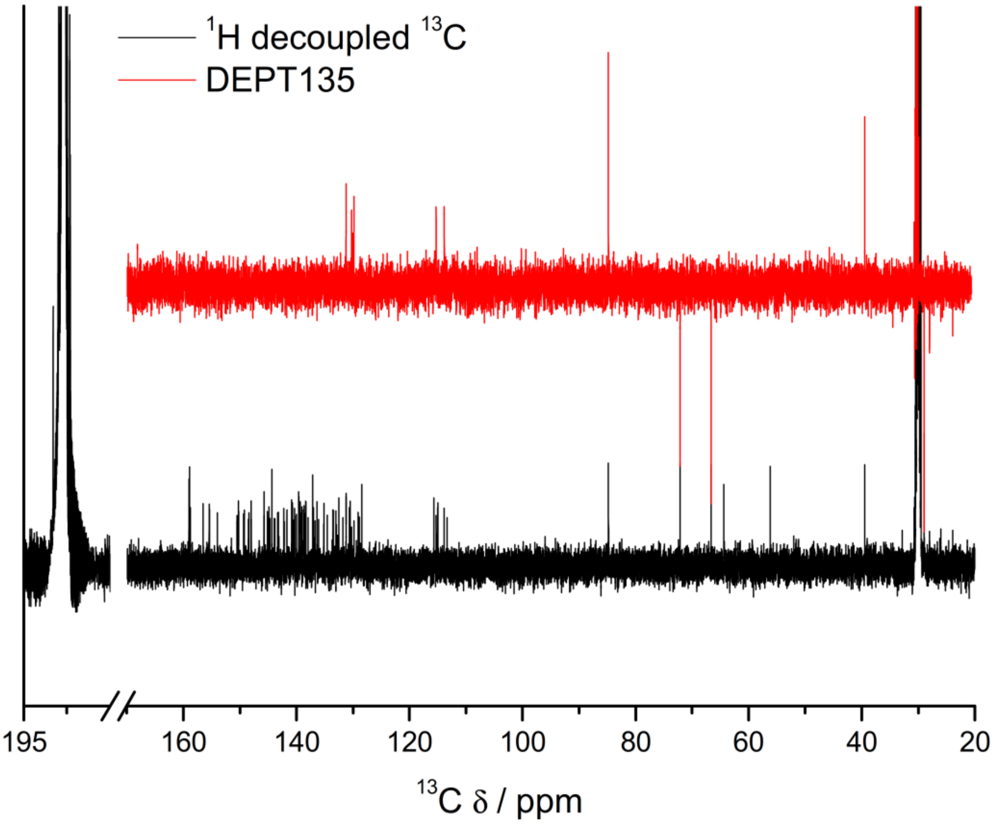

Figure S3. ^{1}H decoupled ^{13}C and DEPT135 NMR spectra of **$Sc_3N@C_{80}$-R-SAc**. Approximately 90 signals are detected at 258 K. Based on DEPT data, 10 of them can be directly assigned to proton-bonded carbons. Carbon signal at 193.3 ppm shows cross-peak to –CO–CH_3 and –S–CH_2 protons and can be thus assigned to the acetyl group.

Table S1. NMR signals of **$Sc_3N@C_{80}$-R-SAc** and their assignment

group	^{1}H δ, ppm	^{13}C δ, ppm (via HSQC)	^{13}C δ, ppm (via HMBC)
**$-CO-CH_3$**	1,71	30,55	-
**$>N-CH_3$**	1,84	39,50	-
**$-CH_{2(a)}-N<$**	2,32	72,13	-
**$-S-CH_2-$**	2,60	28,98	-
**>N-CH<**	2,95	84,83	-
**$-O-CH_2-$**	3,43	66,62	-
**$-CH_{2(b)}-N<$**	3,64	72,13	-
**aromatic**	6,14	113,84	-
	6,44	131,23	-
	6,45	115,31	-
	7,20	129,86	-
**aromatic (tert. >C-O-)**	-	-	158,92
**aromatic (tert. >C-CH-)**	-	-	128,60
**sp^3 – cage 1**	-	-	57,31
**sp^3 – cage 2**	-	-	63,30
**$-S-C=O\_CH_3$**	-	-	193,30

Synthesis of $DySc_2N@C_{80}$-R-SAc

$DySc_2N@C_{80}$ (0.5 mg, 0.4 x 10^{-3} mmol), N-methyl glycine (0.5 mg, 6 x 10^{-3} mmol), *p*-[(2-acetylthio)ethoxy]benzaldehyde (4.6 mg, 0.02 mmol) and *o*-dichlorobenzene (2 mL) were introduced in an oven-dried 25 mL three-neck round-bottom flask, under a dinitrogen atmosphere. The solution was heated to 140°C for 1 hr. The solution was then cooled to room temperature, the solvent was evaporated, and the residue was purified by semi-preparative HPLC with a Buckyprep column (10 × 250 mm), eluting with toluene (flow rate: 1.5 mL min^{-1}). The brown compound **$DySc_2N@C_{80}$-R-SAc** was collected at the retention time of 25 min. HPLC retention time of **$DySc_2N@C_{80}$-R-SAc** is considerably shorter than the retention of the pristine $DySc_2N@C_{80}$ (60 min in the same conditions), which ensures that the pristine fullerene is not present in the collected fraction and the ion of **$DySc_2N@C_{80}^-$** observed in the MALDI mass-spectrum is due to the fragmentation of the derivative during the laser evaporation and ionization. MALDI m/z 1479.081

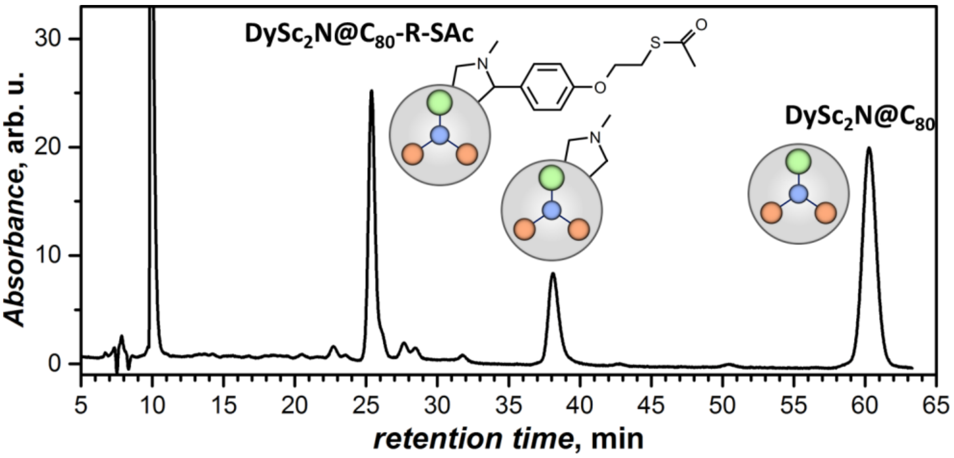

Figure S4a. HPLC trace of the reaction mixture after 1 hour. The peak at 25–26 min is **$DySc_2N@C_{80}$-R-SAc**. Pristine (unreacted) $DySc_2N@C_{80}$ elutes at 60–61 min.

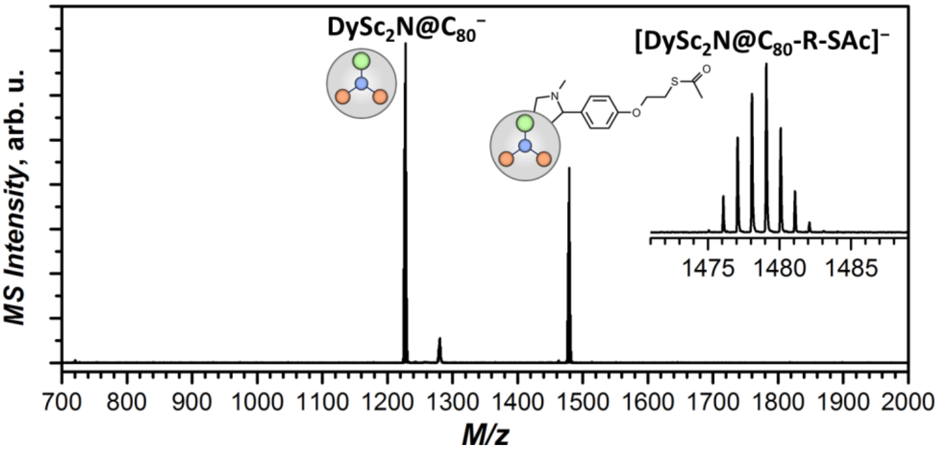

Figure S4b. MALDI-TOF mass-spectrum of the fraction collected at 25–26 min, proving that it is pure **$DySc_2N@C_{80}$-R-SAc**. The peak of $DySc_2N@C_{80}^-$ (1227.9 m/z) and $DySc_2N@C_{80}$-C_3H_7N (1284.9 m/z) are due to the fragmentation of **$DySc_2N@C_{80}$-R-SAc** under laser evaporation conditions; since retention time of pristine $DySc_2N@C_{80}$ and $DySc_2N@C_{80}$-C_3H_7N are much longer (Fig. S4a), their presence in the HPLC-separated sample can be excluded.

Synthesis of $Dy_2ScN@C_{80}$-R-SAc

An oven-dried 25 mL three-neck round-bottom flask was charged with $Dy_2ScN@C_{80}$ (0.5 mg, 0.37 x 10^{-3} mmol), N-methyl glycine (0.5 mg, 5 x 10^{-3} mmol) and *p*-[(2-acetylthio)ethoxy]benzaldehyde (4.2 mg, 0.018 mmol) under a dinitrogen atmosphere. *o*-dichlorobenzene (2.5 mL) was introduced into the flask via a syringe, and the solution was heated to 140°C for 1 hr. The solution was cooled to room temperature, the solvent was evaporated, and the residue was purified by semi-preparative HPLC with a Buckyprep column (10 × 250 mm), eluting with toluene (flow rate: 1.5 mL min^{-1}). The brown compound **$Dy_2ScN@C_{80}$-SAc** was collected at the retention time of 25 min. HPLC retention time of **$Dy_2ScN@C_{80}$-R-SAc** is considerably shorter than the retention of the pristine $Dy_2ScN@C_{80}$ (59 min in the same conditions), which ensures that the pristine fullerene is not present in the collected fraction and the ion of **$Dy_2ScN@C_{80}$**$^-$ observed in the MALDI mass-spectrum is due to the fragmentation of the derivative during the laser evaporation and ionization. MALDI m/z 1598.278

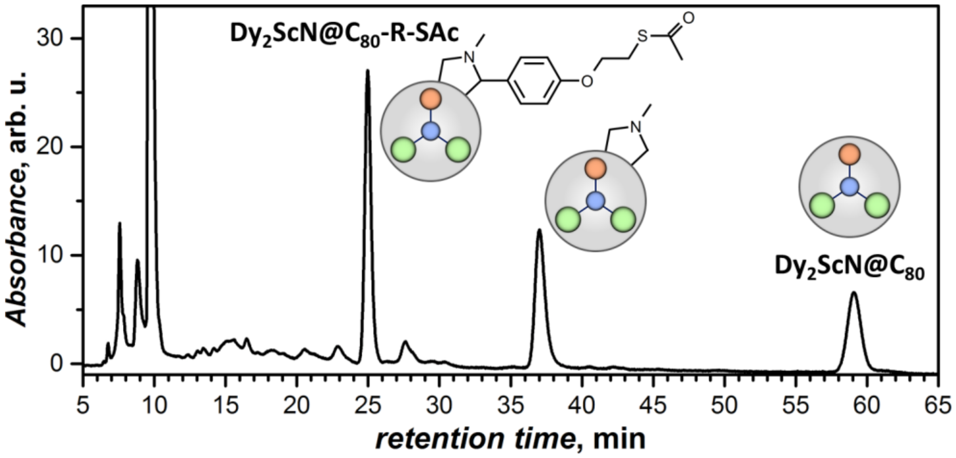

Figure S5a. HPLC trace of the reaction mixture after 1 hour. The peak at 25 min is **$Dy_2ScN@C_{80}$-R-SAc**. Pristine (unreacted) $Dy_2ScN@C_{80}$ elutes at 59 min.

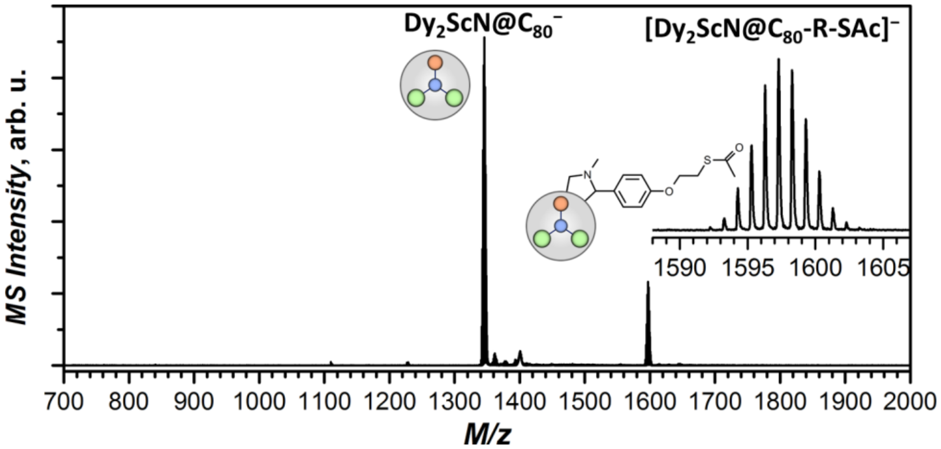

Figure S5b. MALDI-TOF mass-spectrum of the fraction collected at 25 min, proving that it is pure **$Dy_2ScN@C_{80}$-R-SAc**. The peak of $Dy_2ScN@C_{80}^-$ (1344.8 m/z) and $Dy_2ScN@C_{80}$-C_3H_7N (1401.9 m/z) are due to the fragmentation of **$Dy_2ScN@C_{80}$-R-SAc** under laser evaporation conditions. Since retention time of pristine $Dy_2ScN@C_{80}$ and $Dy_2ScN@C_{80}$-C_3H_7N are much longer (Fig. S5a), their presence in the HPLC-separated sample can be excluded.

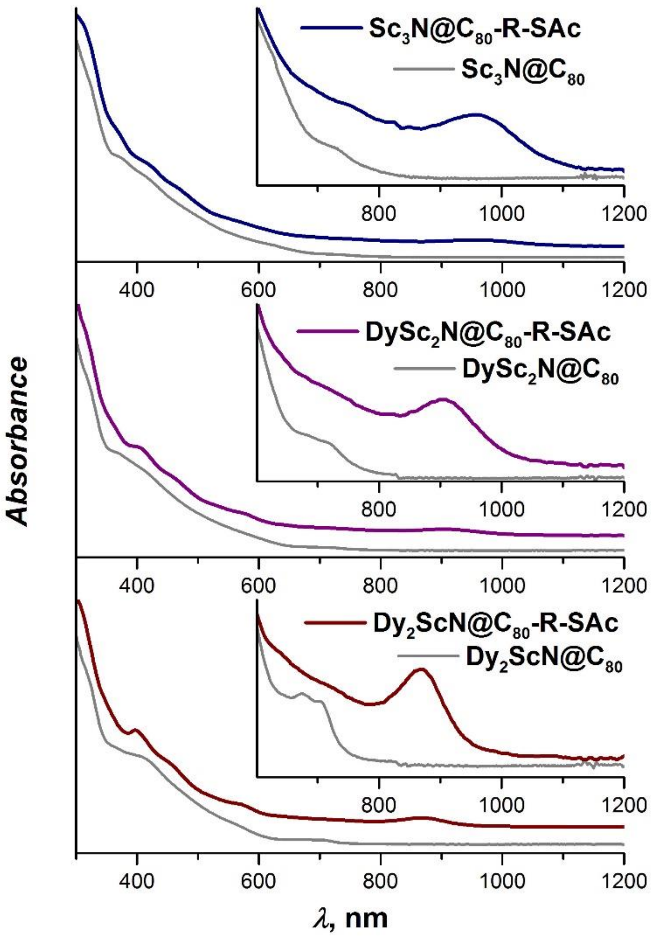

Figure S6. UV-vis-NIR absorption spectra of pristine fullerenes and **$M_3N@C_{80}$-R-SAc** derivatives. The insets show magnification of the NIR range.

SQUID magnetometry of $DySc_2N@C_{80}$-R-SAc

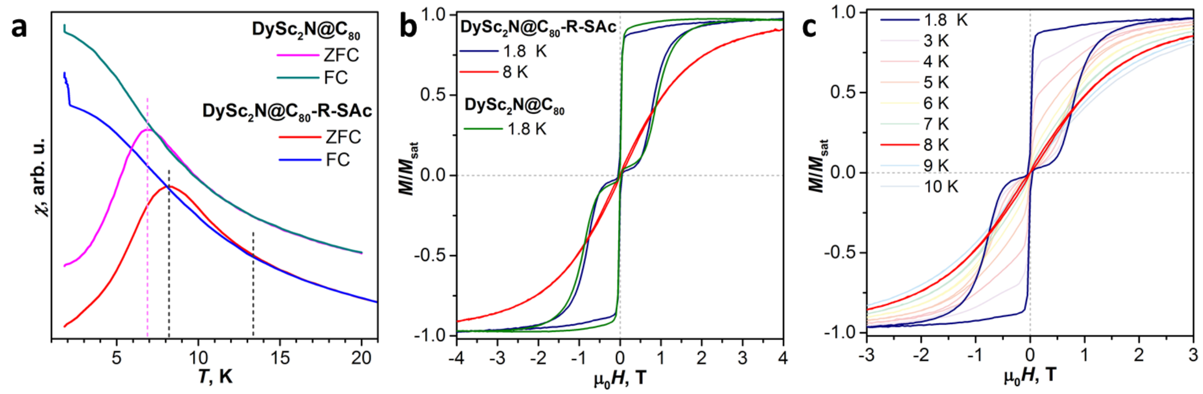

Figure S7. SQUID magnetometry studies of **$DySc_2N@C_{80}$-R-SAc**. (a) Blocking temperature (T_B) determination from the measurement of magnetic susceptibility for field-cooled (FC) and zero-field cooled (ZFC) sample; for comparison analogous measurements with the pristine $DySc_2N@C_{80}$ are also shown (temperature sweep rate 5 K/min, field 0.2 T, curves for two compounds are off-set for clarity); note that the peak in χ_{ZFC} is found at 8.2 K, but considerable deviations of χ_{ZFC} and χ_{FC} curves are seen up to 13–14 K. (b) Magnetic hysteresis curves of **$DySc_2N@C_{80}$-R-SAc** measured at 1.8 K and 8 K compared to the curve measured for $DySc_2N@C_{80}$; sweep rate 2.9 mT/s. (c) Magnetic hysteresis curves of **$DySc_2N@C_{80}$-R-SAc** measured at different temperatures with the sweep rate of 2.9 mT/s (0.18 T/min).

SQUID magnetometry of $Dy_2ScN@C_{80}$-R-SAc

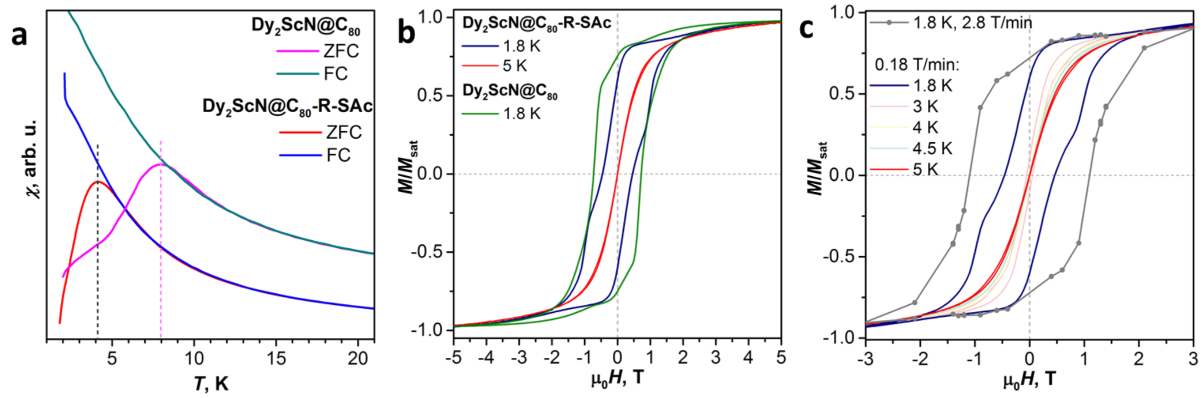

Figure S8. SQUID magnetometry studies of **$Dy_2ScN@C_{80}$-R-SAc**. (a) Blocking temperature (T_B) determination from the measurement of magnetic susceptibility for field-cooled (FC) and zero-file cooled (ZFC) sample; for comparison analogous measurements were the pristine $Dy_2ScN@C_{80}$ are also shown (temperature sweep rate 5 K/min, field 0.2 T, curves for two compounds are off-set for clarity). (b) Magnetic hysteresis curves of **$Dy_2ScN@C_{80}$-R-SAc** measured at 1.8 K and 5 K compared to the curve measured for $Dy_2ScN@C_{80}$; sweep rate 2.9 mT/s. (c) Magnetic hysteresis curves of **$Dy_2ScN@C_{80}$-R-SAc** measured at different temperature with the sweep rate of 2.9 mT/s (0.18 T/min); also shown is the curve measured at 1.8 K with the sweep rate of 2.8 T/min.

Self-assembly from solution and XPS characterization

The procedure for the growth of fullerene SAM was adopted from Refs. 2, where it was developed for C_{60} derivatives. Freshly synthesized Au(111)|mica substrates (PHASYS) were immersed into *o*-DCB/EtOH/H_2SO_4 (200/20/1) solution of the EMFs derivatives (concentration ca 13 µmol/L) for 90 min. Then, the substrate was taken out, washed with excess of *o*-DCB and ethanol and then dried under nitrogen stream. Fresh samples were prepared before each measurement (i.e. XMCD and XPS studies at different research facilities were performed for different samples but prepared following the same procedure).

XPS studies were performed at Russian-German beamline of BESSY II. Figure S9a shows overview spectra in the binding energy range up to 350 eV (including Au-4f, S-2p, and C-1s features), whereas Fig. S9b shows the S-2p range with fitting of the peaks. Results of the fitting are summarized in Table S2. Both **$DySc_2N$-SAM** and **Dy_2ScN-SAM** samples exhibit features of the Au-bonded sulfur at 161.1. eV confirming chemisorption of fullerenes. However, the signals of the non-bonded sulfur at 162-163 eV and oxidized sulfur at 169 eV are also detected. The oxidized sulfur likely originates from the traces of the sulfuric acid used in the deposition procedure. This is confirmed by the overall carbon to sulfur ratio of 96.5 to 3.5 for **$DySc_2N$-SAM** and 95.8 to 4.2 for **$DySc_2N$-SAM** showing an excess of sulfur over carbon (theoretical value for derivatized fullerene molecule is 91:1). Thus, there is a significant amount of sulfur, which is not bonded to fullerene.

We could not detect Dy-4d signals in the XPS spectra, which are expected at 155 eV and should be ca 3 time less intense than S-2p signal (considering attenuation factor of the carbon cage, assumed to be 0.55 as in graphene). However, the presence of Dy in the sample is unambiguously confirmed by XAS data (see below).

Similar spectra to those shown in Fig. S9b were observed by Tour et al. in their study of C_{60}-SAMs prepared from C_{60} functionalized with thiol and thioacetate groups.[2] The authors also found a combination of signals from oxidized, bonded and non-bonded sulfur. They concluded that combination of bonded and non-bonded sulfur originates from the head-to-tail arrangement of the molecules on the surfaces since ellipsometry analysis proved formation of monolayers in all cases. It is likely that similar effect takes place in this work with $DySc_2N@C_{80}$ and $Dy_2ScN@C_{80}$ SAMs. Note also that the deposited layers in our work were thoroughly washed with *o*-DCB and ethanol after SAM formation, and it is very unlikely that a physisorbed multilayer might withstand this procedure. Finally, the overall Dy-$M_{4,5}$ signals observed in XAS spectra (see below) are also consistent with the coverage close to but below than a monolayer.

2. Y. Shirai, L. Cheng, B. Chen, J. M. Tour, *J. Am. Chem. Soc.* **2006**, *128*, 13479-13489

Table S2. Fitting of XPS spectra in the range of S-2p bands

Type		E_{bind} $2p_{3/2}/2p_{1/2}$, eV	Area, %	Total signal, %
**$DySc_2N$-SAM**				
S-I (blue)	S-Au	161.1/162.2	13.26/6.63	20.0
S-II (cyan)	S-Ac/SH	162.0/163.1	10.68/5.34	16.0
S-III (pink)	S-Ac/SH	163.35/164.45	9.66/4.83	14.4
S-IV (yellow)*	SO_x	168.8		49.6
**Dy_2ScN-SAM**				
S-I (blue)	S-Au	161.1/162.2	10.8/5.4	16.2
S-II (cyan)	S-Ac/SH	162.0/163.1	17.0/8.5	25.5
S-III (pink)	S-Ac/SH	163.35/164.45	12.55/6.3	18.8
S-IV (yellow)*	SO_x	168.8; 166.5	37.3, 2.2	39.5

* as the signal of oxidized sulfur (S-IV) is too broad, so it was fitted by a single peak

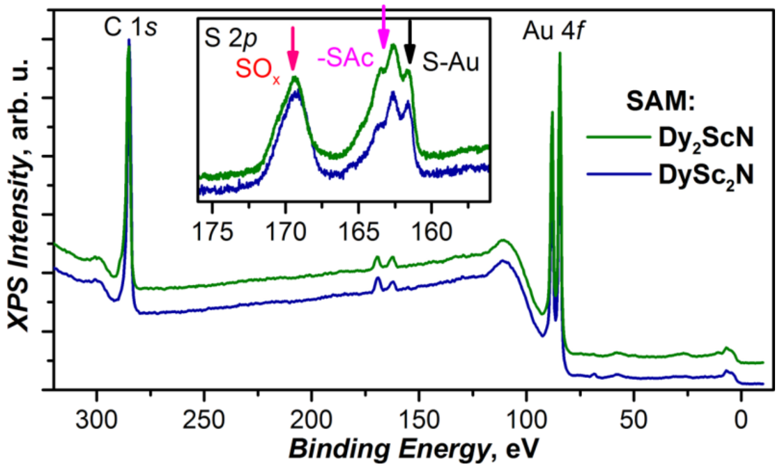

Figure S9a. XPS spectra of **DySc$_2$N-SAM** and **Dy$_2$ScN-SAM**.

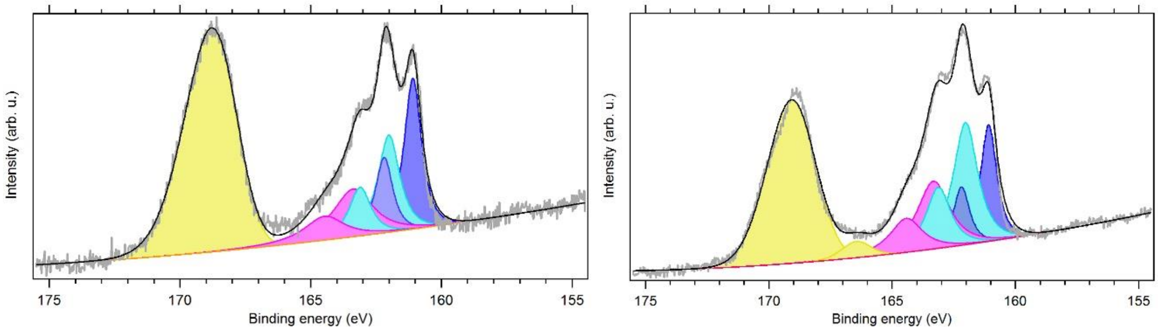

Figure S9b. XPS spectra of **DySc$_2$N-SAM** (left) and **Dy$_2$ScN-SAM** (right) in the range of S-2p features with fitting of the bands.

Technical details of polarized XAS studies

X-ray absorption measurements with linear or circular polarized light were performed at beamlines X-Treme (Swiss Light Source, Paul-Sherrer Institute), UE46_PGM-1 (BESSY II), and DEIMOS (synchrotron SOLEIL). In all cases, the total electron yield detection mode was used. To take into account the variation of the X-ray beam intensity, the current from the sample was referred to the current from the gold grid. Then, each measured spectrum was normalized to the intensity of the pre-edge signal, and then the broad background was subtracted. As a result of this procedure, the XAS signal intensity reported in the paper corresponds to the normalized increase of the XAS due to the specific absorption at the Dy-$M_{4,5}$ edge over the background signal and hence can be directly compared for different incidence angles and for different samples measured at different facilities. Intensities of the vertically and horizontally linear polarization are denoted as I^{vert} and I^{horiz}, whereas those for clockwise and anti-clockwise circular polarization as I^{+} and I^{-}. Non-polarized XAS intensity is defined as XAS = ($I^{\mathrm{vert}} + I^{\mathrm{horiz}}$) or XAS = ($I^{+} + I^{-}$), XLD intensity is ($I^{\mathrm{horiz}} - I^{\mathrm{vert}}$), whereas XMCD intensity is ($I^{-} - I^{+}$). When XMCD and XLD intensity are plotted in %, XMCD or XLD signal is divided by the XAS intensity at its maximum.

In the measurement of magnetization curves, I^{+} or I^{-} intensity during each magnetic field sweep was measured at fixed energies corresponding to the maximum of XMCD signal and to the pre-edge energy. In the magnetic hysteresis and angular dependence plots, the ($I^{-} - I^{+}$) values are divided by the ($I^{+} + I^{-}$) sum, giving normalized XMCD, XMCD/XAS_{max}. Note that in the total electron yield mode, the current measured in small magnetic fields varies strongly with the field, which results in unrealistic, strongly oscillating XMCD/XAS_{max} values near zero field during the field sweep.

XAS studies of Dy_2ScN-SAM

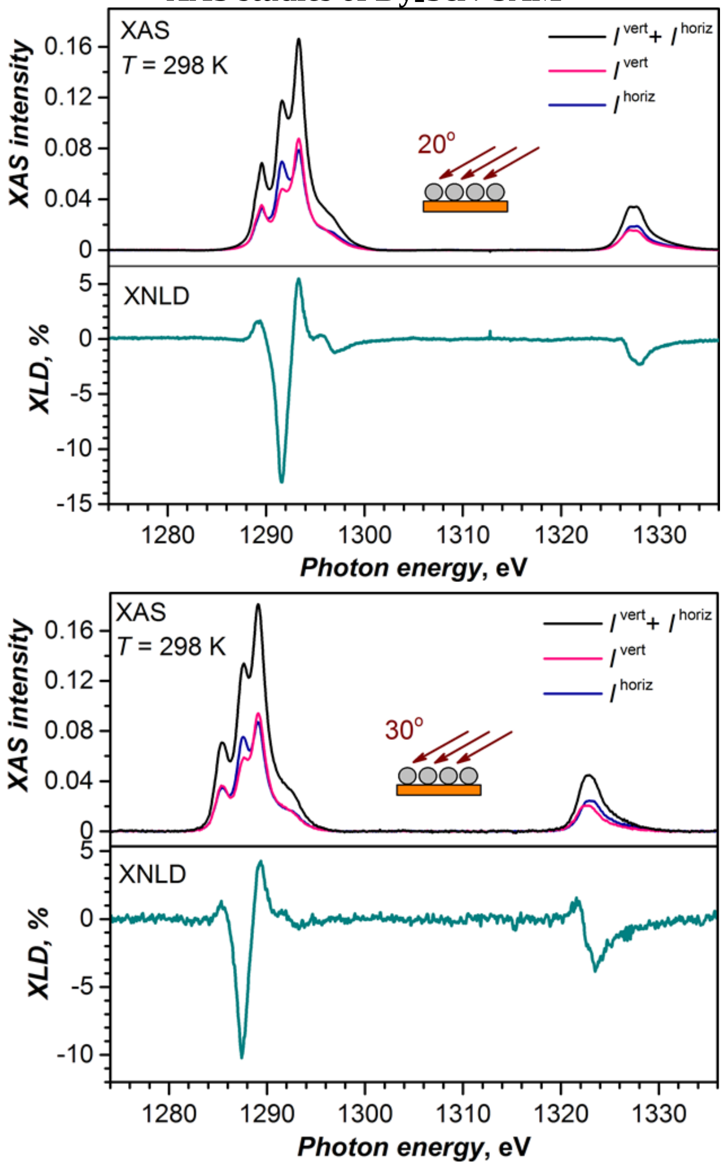

Figure S10. Room temperature XNLD spectra of **Dy_2ScN-SAM** samples measured at BESSY II (upper panel) and at SLS (lower panel), H = 0.1 T. XAS intensity was normalized to the pre-edge absorption, and then the baseline was subtracted. Similar XAS intensity in two panels indicate that the surface coverage in two samples is almost identical. XNLD intensity in the upper panel is somewhat higher, but it may be caused by a smaller angle (20º) which could be achieved in the measurements at BESSY.

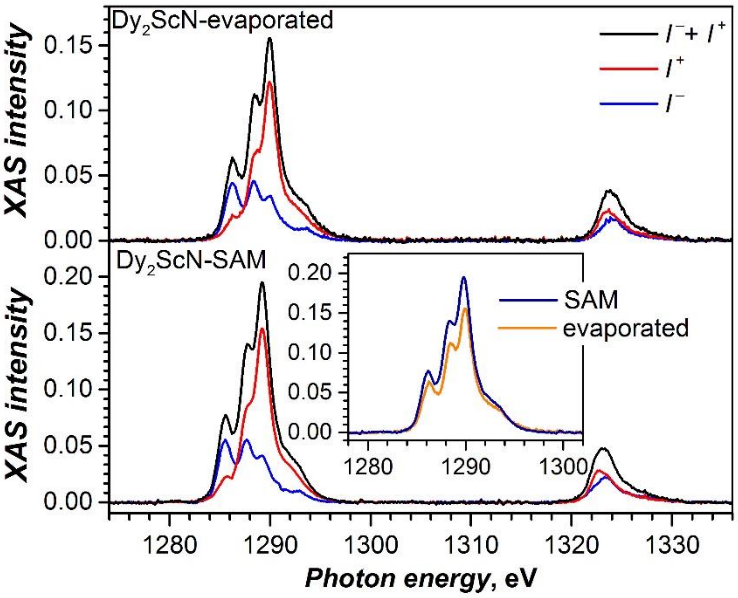

Figure S11. Low-temperature XAS/XMCD spectra of evaporated $Dy_2ScN@C_{80}$ (ca 0.5 ML) and **Dy_2ScN-SAM** measured at SLS (lower panel), H = 6.5 T. The inset shows comparison of XAS spectra at the Dy-M_5 edge. XAS intensity is normalized to the pre-edge absorption, and then the baseline is subtracted. Higher intensity of the **Dy_2ScN-SAM** sample indicates a higher coverage, but still in the monolayer regime (note also that one derivatized molecule occupies more space than the pristine fullerene). Thus, we can conclude that **Dy_2ScN-SAM** sample has a surface coverage close to 1 ML.

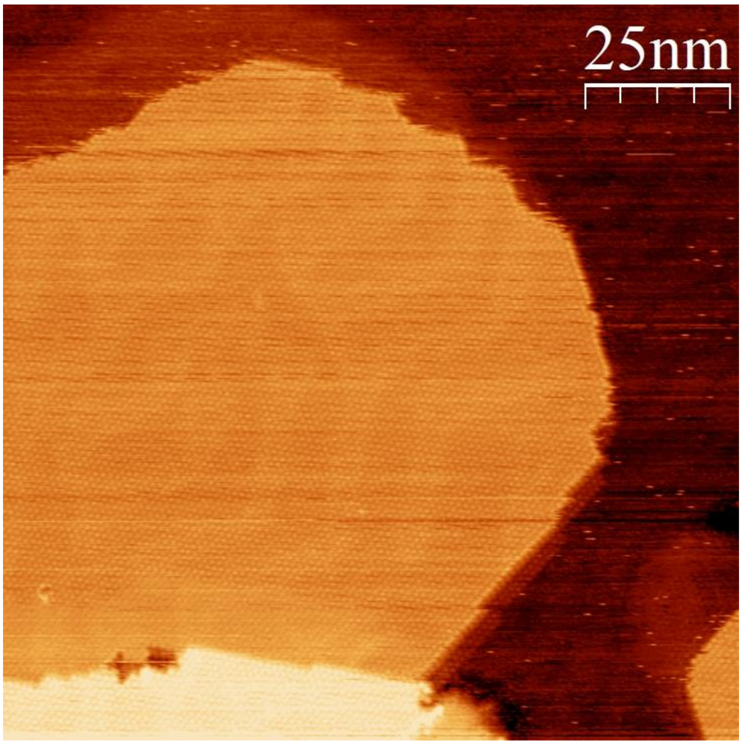

Figure S12. Room-temperature STM constant current topography image (tunneling current 300 pA, surface 125x125 nm, bias 2 V) of a monolayer island in the evaporated $Dy_2ScN@C_{80}$ submonolayer on Au(111) measured *in situ* before XAS studies at the X-Treme beamline (SLS, PSI).

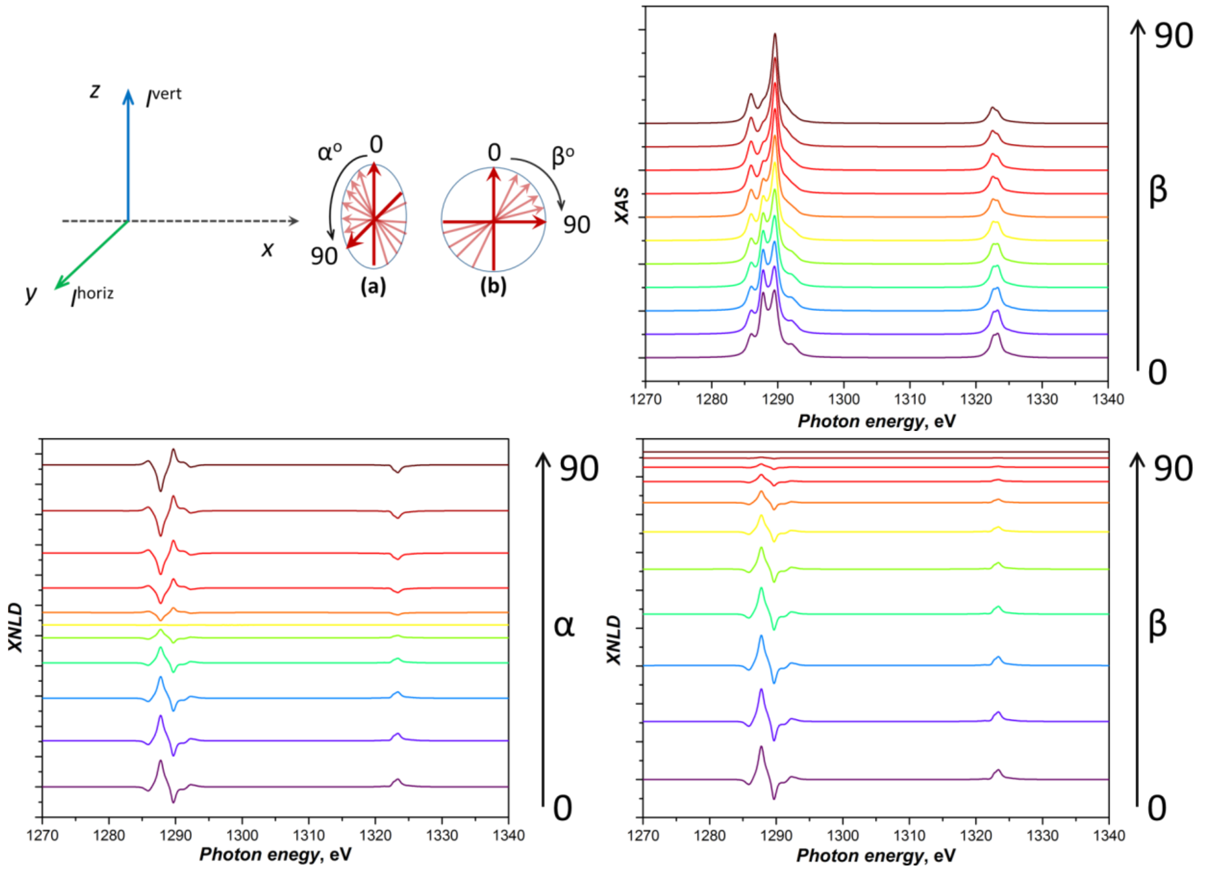

Figure S13. XAS and XNLD spectra simulated for different orientations of Dy–N fragment versus the beam. Upper left figure shows "experimental" geometry, with the beam running parallel to the surface. Dy–N fragment (visualized as a red arrow) is rotated from vertical orientation (0°) to a horizontal one (90°) along two directions designated as α and β. Upper left figure shows XAS spectra simulated for different β from 0° to 90° (the XAS spectrum is not changing with α). Note that the spectrum for the vertical orientation of the moment would show two intense peaks of equal intensity, whereas compete in-plane orientation with β = 90° would have XAS spectrum with a single intense line. At the same time, the spectrum at α = 90° is the same as for β = 0°. Thus, predominant vertical orientation of Dy–N fragment would result in the two-peak pattern. In-plane orientation would show an average situation because of different in-plane orientations of Dy–N bonds. The experimental XAS spectra show an average situation indicating that there is no preference for the vertical orientation of Dy–N bonds. With variation of α, XNLD spectra change the sign (XNLD vanishes at α = 45°), whereas with variation of β, the XNLD intensity decreases to zero at β = 90°. Again, averaged situation is observed in experimental spectra. It is more difficult to make a conclusion on the other orientations since it would require simulations for multiple random orientations of the molecules. However, angular dependence of XMCD signal shows that in-plane orientations are more preferable for magnetic moments (hence Dy–N bonds, because orientation of magnetic moments coincides with that of the Dy–N bonds).

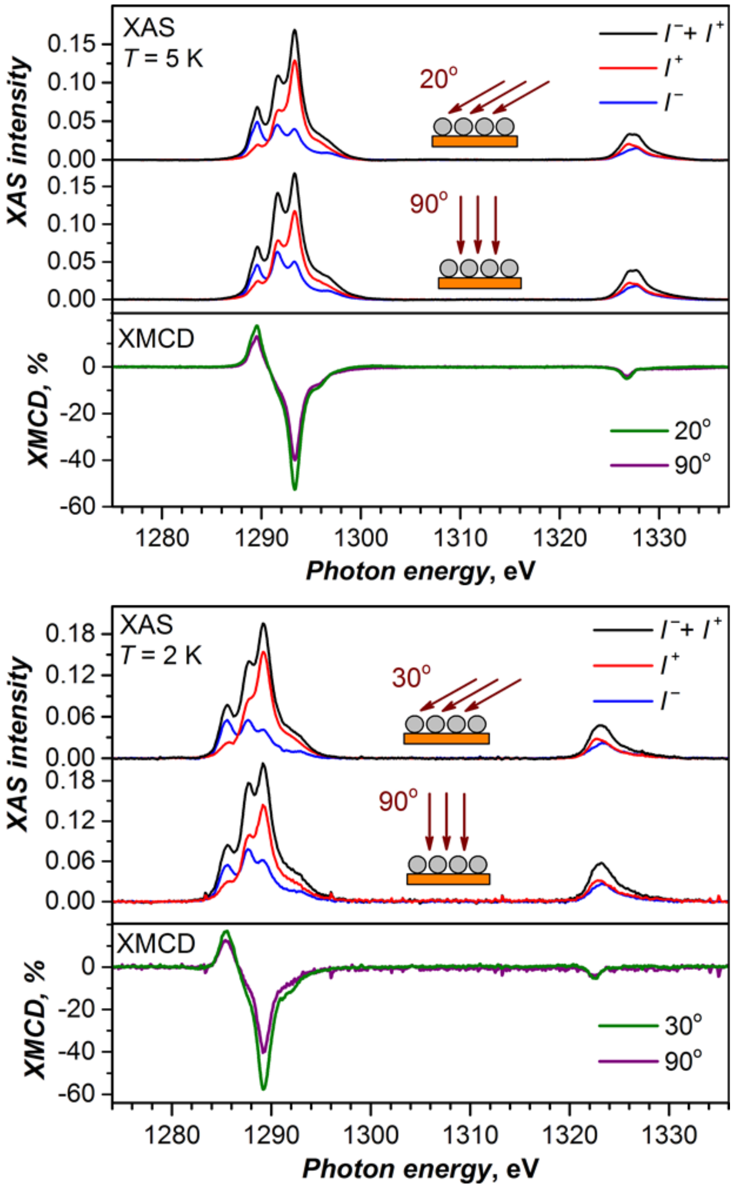

Figure S14. Low-temperature XMCD spectra of **Dy_2ScN-SAM** samples measured at BESSY II (upper panel) and at SLS (lower panel), H = 6.5 T. XAS intensity was normalized to the pre-edge absorption, and then the baseline was subtracted. Similar XAS intensity in two panels indicate that the surface coverage in two samples is almost identical.

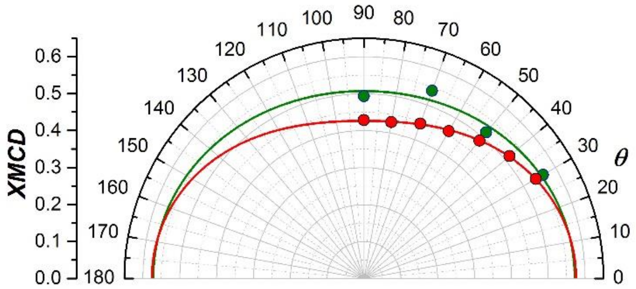

Figure S15. Angular dependence of XMCD/XAS intensity measured for **Dy_2ScN-SAM** (red dots) and evaporated $Dy_2ScN@C_{80}$ submonolayer (green dots). Lines are fits with the function $XMCD/XAS = C_1 \cos^2(\theta) + C_2$

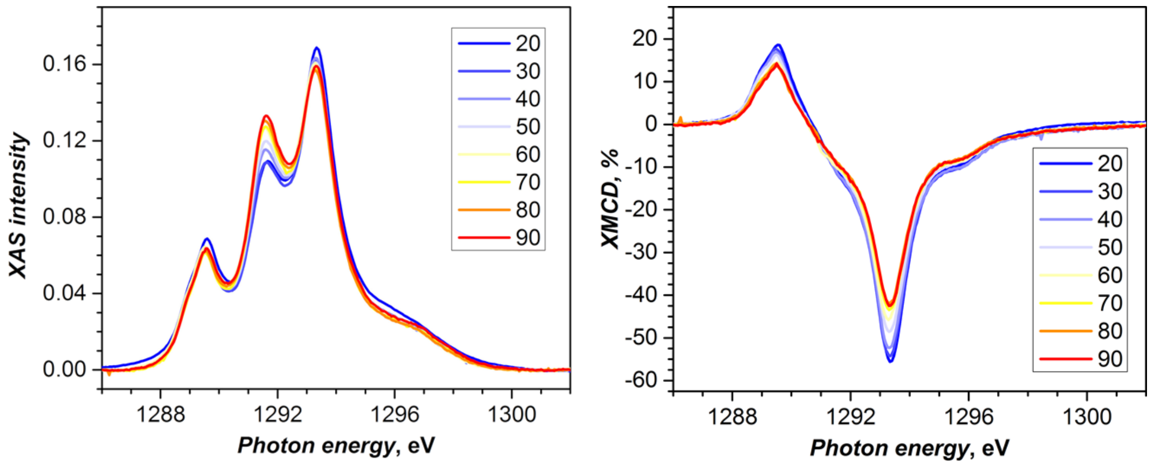

Figure S16. XAS (left) and XMCD (right) spectra of **Dy_2ScN-SAM** measured at the Dy-M_5 edge at different incidence angles, T = 5 K, H = 6 T.

XAS studies of $DySc_2N$-SAM and $DySc_2N@C_{80}$ multilayer

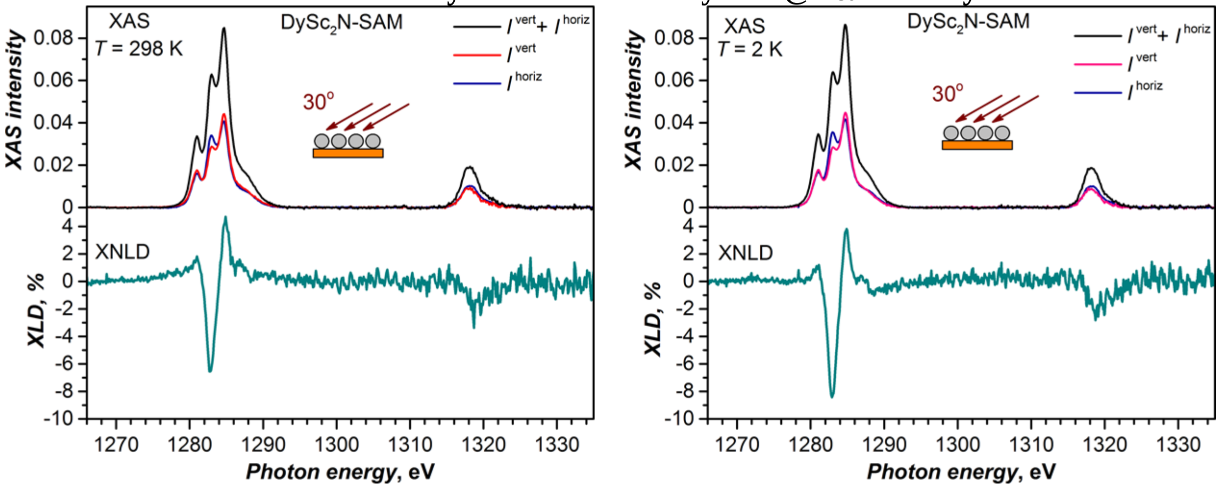

Figure S17. XAS and XNLD spectra of **$DySc_2N$-SAM** measured at room temperature (left) and at 2 K (right), H = 0.1 T. Note that XNLD intensity is slightly increased at low temperature. Note also that normalized XAS intensity of **$DySc_2N$-SAM** at the Dy-M_5 edge is twice lower than that of **Dy_2ScN-SAM** (Figure S10), which shows that similar coverage is obtained for two SAMs.

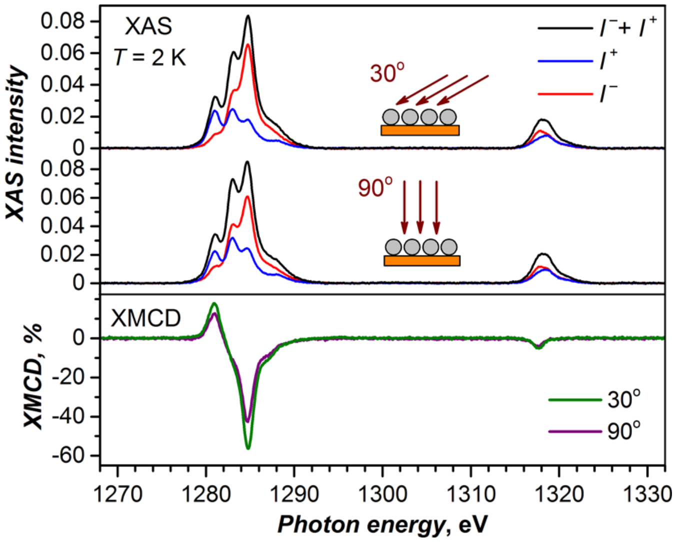

Figure S18. XAS and XMCD spectra of **$DySc_2N$-SAM** measured at 2 K with two incidence angles, H = 6 T.

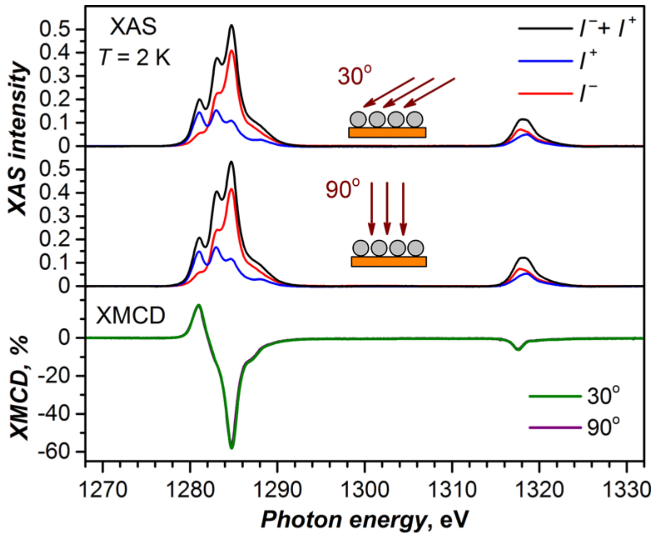

Figure S19. XAS and XMCD spectra of evaporated $DySc_2N@C_{80}$ multilayer measured at 2 K with two incidence angles, H = 6 T.

STM characterization of $DySc_2N@C_{80}$ multilayer

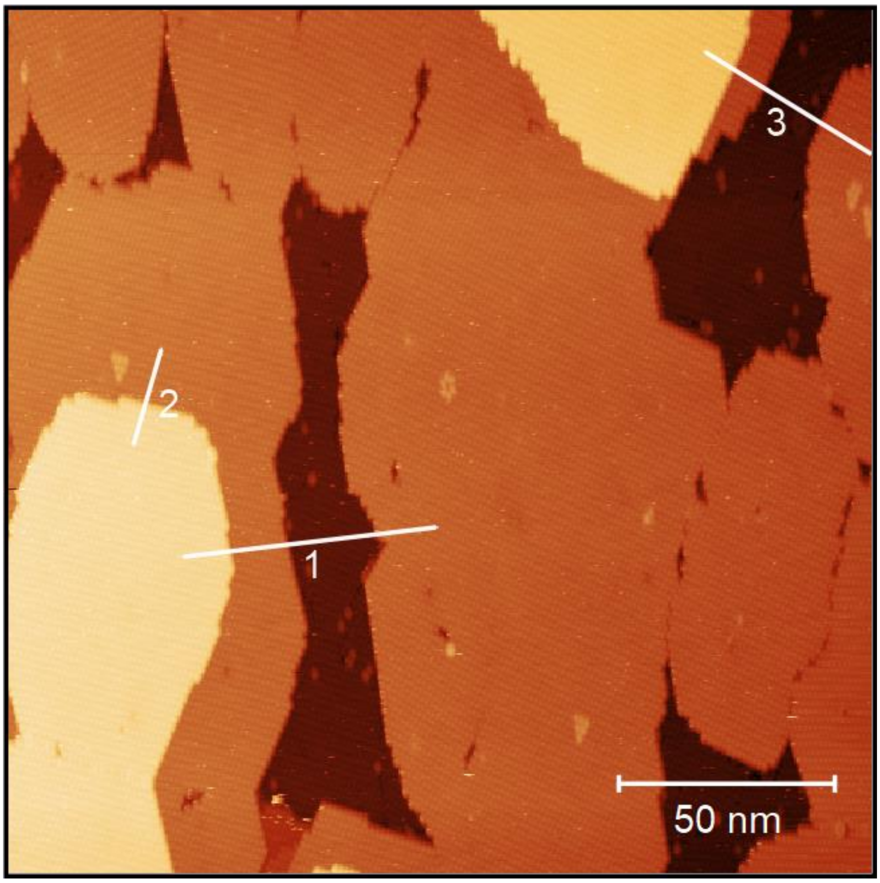

Figure S20a. Room-temperature STM characterization of the $DySc_2N@C_{80}$ multilayer evaporated onto Au(111) crystal. 200 x 200 nm topography (flattening algorithm applied), tunneling current 0.1 nA, bias 2 V. Evaporation time 7 min 30 sec at 525ºC. Height profiles along the lines **1**, **2**, and **3** are shown in Figure 22b. Note that bare Au surface is not seen, indicating that there is no less than 3 MLs of fullerenes (and likely more) in this film.

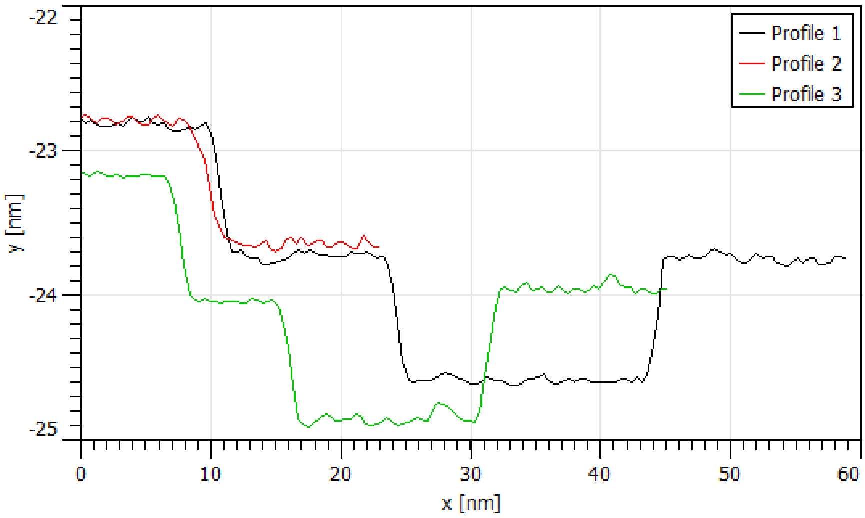

Figure S20b. Height profiles measured along the lines **1**, **2**, and **3** in Fig. S22a. All steps are of 0.8 nm, corresponding to the fullerene size.

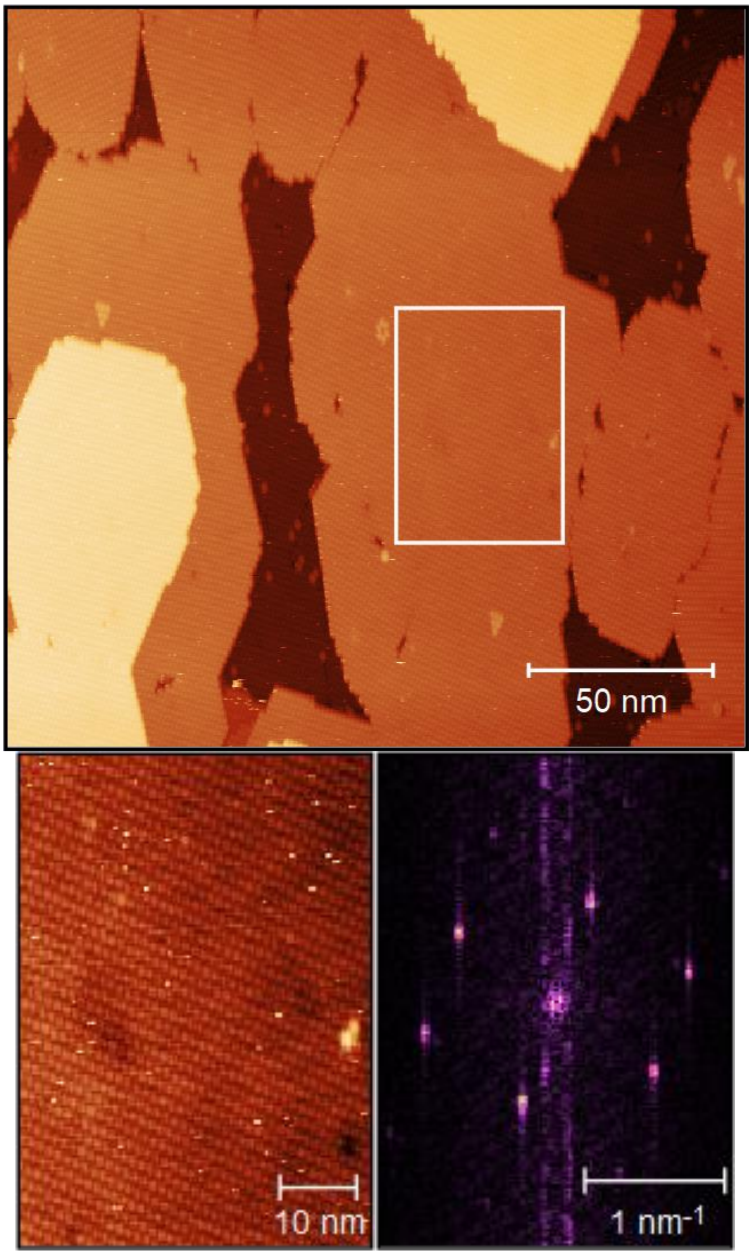

Figure S21. Upper row: Room-temperature STM topography of the $DySc_2N@C_{80}$ multilayer evaporated onto Au(111) crystal (the same as in S22a) highlighting the fragment, for which Fourier-transform is perfomed (lower row). Disrtortion of the hexagonal patterrn in the Fourier map indicates the drift in the measurement (also seen in the magnifiaton of the topograpy image).

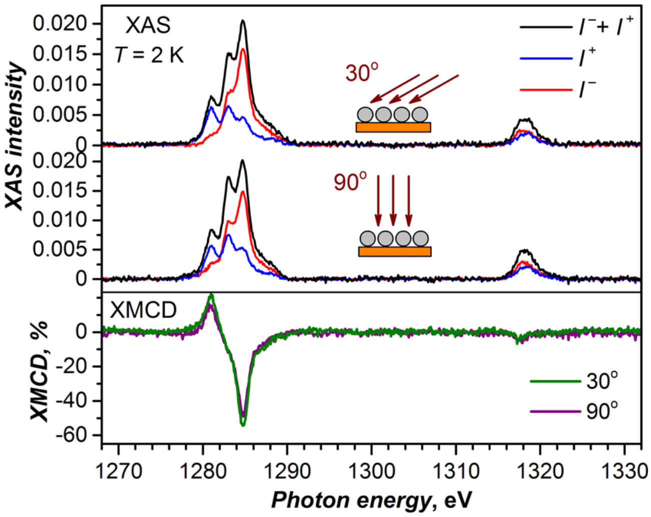

Figure S22. XAS and XMCD spectra of mixed **$DySc_2/Sc_3N$-SAM** measured at 2 K with two incidence angles, H = 6 T.

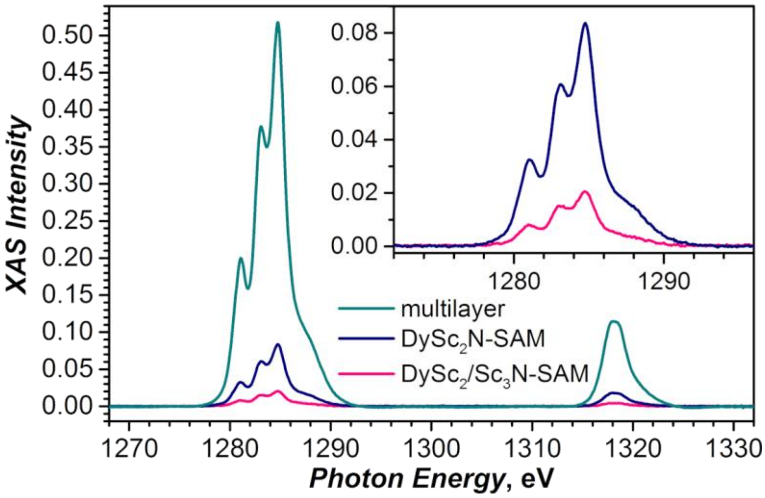

Figure S23. XAS spectra of evaporated $DySc_2N@C_{80}$ multilayer, **$DySc_2N$-SAM**, and **$DySc_2/Sc_3N$-SAM** at the Dy-$M_{4,5}$ edges measured at 2 K, incidence 30°. Intensity ratio multilayer : **$DySc_2N$-SAM** is 6:1, intensity ratio **$DySc_2N$-SAM** : **$DySc_2/Sc_3N$-SAM** is 1:0.24.

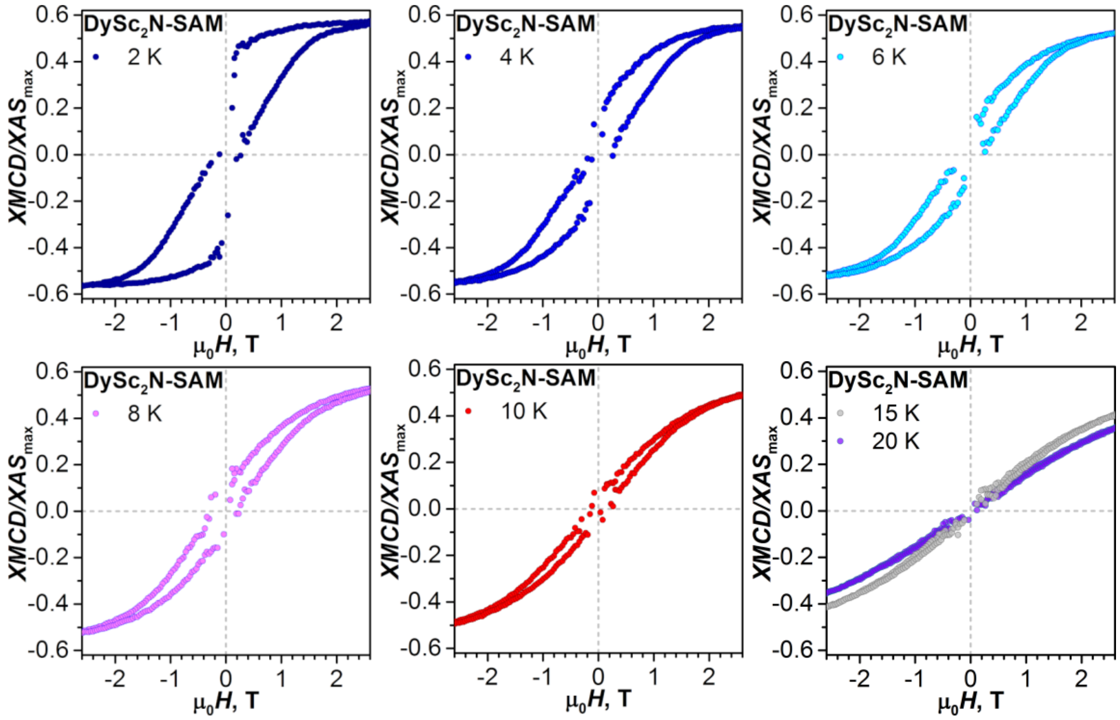

Figure S24. Magnetization curves of **$DySc_2N$-SAM** measured by XMCD at different temperatures. Sweep rate 2 T/min, incidence – 30º.
